# Hydrochar and Arbuscular Mycorrhizal Fungi Improve Soil Properties and Plant Growth in Coal Gangue-Amended Sandy Soil

**DOI:** 10.3390/plants15203201

**Published:** 2026-10-19

**Authors:** Huiping Song, Rui Zhang, Junjie Zhang, Zhanjin Xue, Xiaohui Wang, Yan Zhao, Zhengjun Feng

**Affiliations:** 1 Institute of Resources and Environmental Engineering, Shanxi University, Taiyuan 030031, China; songhp@sxu.edu.cn (H.S.); zhangrui19@sxu.edu.cn (R.Z.); 17838458261@163.com (J.Z.); xuezhanjin@sxu.edu.cn (Z.X.); 2 Changzhi Key Laboratory for Testing and Application of Solid Waste New Materials, Changzhi 046000, China; 3 Anhui Provincial Academy of Eco-Environmental Science Research, Hefei 230061, China; wxh0712@163.com (X.W.); yanzhao.ahhky@gmail.com (Y.Z.)

**Keywords:** arbuscular mycorrhizal fungi, coal gangue, hydrochar, plant growth, sandy soil, soil restoration

## Abstract

This study evaluated hydrochar and arbuscular mycorrhizal fungi (AMF) for improving coal gangue-amended sandy soil and plant performance. A ryegrass (*Lolium perenne* L.) pot experiment compared corncob, corn straw, and rice husk hydrochars applied at 2–8%. Selected 8% hydrochars were then evaluated in a four-month alfalfa (*Medicago sativa* L.) rhizobox experiment to examine soil and plant responses across a 30 cm soil profile and the additional response to AMF inoculation. Hydrochar significantly affected soil nutrient status and all seven ryegrass growth and root traits, with 8% rice husk hydrochar (RHH8) showing the strongest overall performance. After cultivation, soil organic matter was 32.85 g kg^−1^ under RHH8 versus 14.81 g kg^−1^ under the control (CK). In the rhizobox experiment, compared with 8% rice husk hydrochar alone (RHH), AMF addition to RHH was associated with higher soil nutrient levels, greater dry-sieved aggregation and water retention, and a greater maximum root length in alfalfa (33.46 vs. 25.68 cm). Total soil concentrations of all eight measured metals were below the GB 15618-2018 agricultural-land risk screening values at the measured endpoints. Overall, RHH performed consistently well, with additional improvements following AMF inoculation. These short-term findings support further evaluation for restoring coal gangue-amended sandy soil.

## 1. Introduction

The area of sandy soil in China has reached 1.68 million km^2^, accounting for 17.58% of the country’s total land area, making China one of the countries with the most severe sandy desertification in the world. Especially in major coal-producing regions such as Shanxi, Shaanxi, and Inner Mongolia, coal gangue is produced in large quantities annually and stored in open piles for long periods [1]. This not only destroys the surface structure and leads to vegetation loss but also, combined with water scarcity and climate change, exacerbates regional land desertification and imposes serious ecological pressure [2]. The management of sandy soils and ecological restoration of mining areas [3] have therefore become important priorities for mitigating land degradation and supporting sustainable development [4,5].

Coal gangue is a mixture of carbonaceous, argillaceous, and sandy shales, mainly composed of Al_2_O_3_, SiO_2_, etc. [6]. It shares some mineral and physicochemical characteristics with natural soil and contains mineral components (e.g., Al, Si) and trace nutrients and can therefore be used as a soil amendment [7]. The similarity in particle structure and physicochemical properties between coal gangue and sandy soil has been demonstrated, providing the possibility for their combined use [8,9]. Previous research by Zhang et al. [10] confirmed that mixing fine coal gangue with sandy soil can fill the large pores of sandy soil and increase the proportion of small pores, transforming the soil texture from sand to sandy loam and, to some extent, improving total porosity and water-holding capacity. Nevertheless, coal gangue alone may not fully alleviate the constraints associated with sandy soils [11]. Its effects on soil hydraulic properties are strongly influenced by particle size and application rate, while excessive inputs of fine particles may reduce soil porosity and hydraulic conductivity [11,12]. In addition, the coarse texture and low organic matter content of sandy soils inherently limit their capacity to retain water and nutrients [13,14]. Consequently, improvements in selected physical properties may not necessarily translate into adequate water and nutrient availability for sustained plant growth [11,13,14]. Complementary amendments are therefore required to address these remaining limitations and provide more favorable conditions for vegetation establishment [13,14].

Hydrochar has received increasing attention as a carbonaceous amendment for soil improvement and plant production [15,16]. Its effects on soil properties and plant growth vary with hydrochar characteristics, application rate, and soil type [16,17,18]. In sandy soils, hydrochar application has been shown to decrease bulk density, modify porosity, increase water retention, and reduce nitrate leaching [19,20]. Hydrochar can also alter soil nutrient availability and plant growth, although these responses are strongly dependent on application rate and soil conditions [17,18,19,21]. Recent evidence further indicates that hydrochar can substantially modify soil hydraulic behavior, including water retention, hydraulic conductivity, and infiltration, with different hydraulic processes responding differently to hydrochar addition [12,20]. Collectively, these findings demonstrate the potential of hydrochar to alleviate water and nutrient limitations in sandy soils, while highlighting the importance of selecting appropriate hydrochar properties and application rates for specific soil conditions [12,16,17,18,19,20,21].

Plant root-mediated processes represent an important biological pathway for sandy soil restoration, as root growth promotes soil aggregation and structural stability and thereby enhances resistance to erosion [22,23]. Root exudates further regulate rhizosphere processes and contribute to soil carbon and nutrient cycling [24,25]. In addition, symbiosis with arbuscular mycorrhizal fungi (AMF) extends the soil volume explored by plants, enhances nutrient acquisition, and improves plant performance under nutrient limitation and drought stress [26,27,28].

Previous studies have investigated coal gangue for substrate reconstruction [11,29], hydrochar for improving sandy soils [12,19,20], and carbonaceous amendments combined with AMF for degraded-soil restoration [30,31,32,33]. However, these approaches remain insufficiently integrated in coal gangue-amended sandy soils. In particular, little is known about how hydrochar feedstock and application rate influence soil–plant responses in this system or whether subsequent AMF inoculation provides additional benefits. The scope of the present study relative to previous restoration studies is summarized in Table S1. To address these questions, we conducted two complementary experiments. A pot experiment first compared three hydrochars applied at 2–8% to evaluate their effects on soil properties and ryegrass growth. Selected hydrochars at 8% were then examined in a rhizobox experiment with alfalfa, in which soil and plant responses were evaluated across soil depths and sampling times, and the additional response to AMF inoculation following rice husk hydrochar amendment was assessed.

## 2. Results

### 2.1. Improvement of Sandy Soil by Hydrochar and Plants

#### 2.1.1. Pot Soil Responses

**Figure 1:**
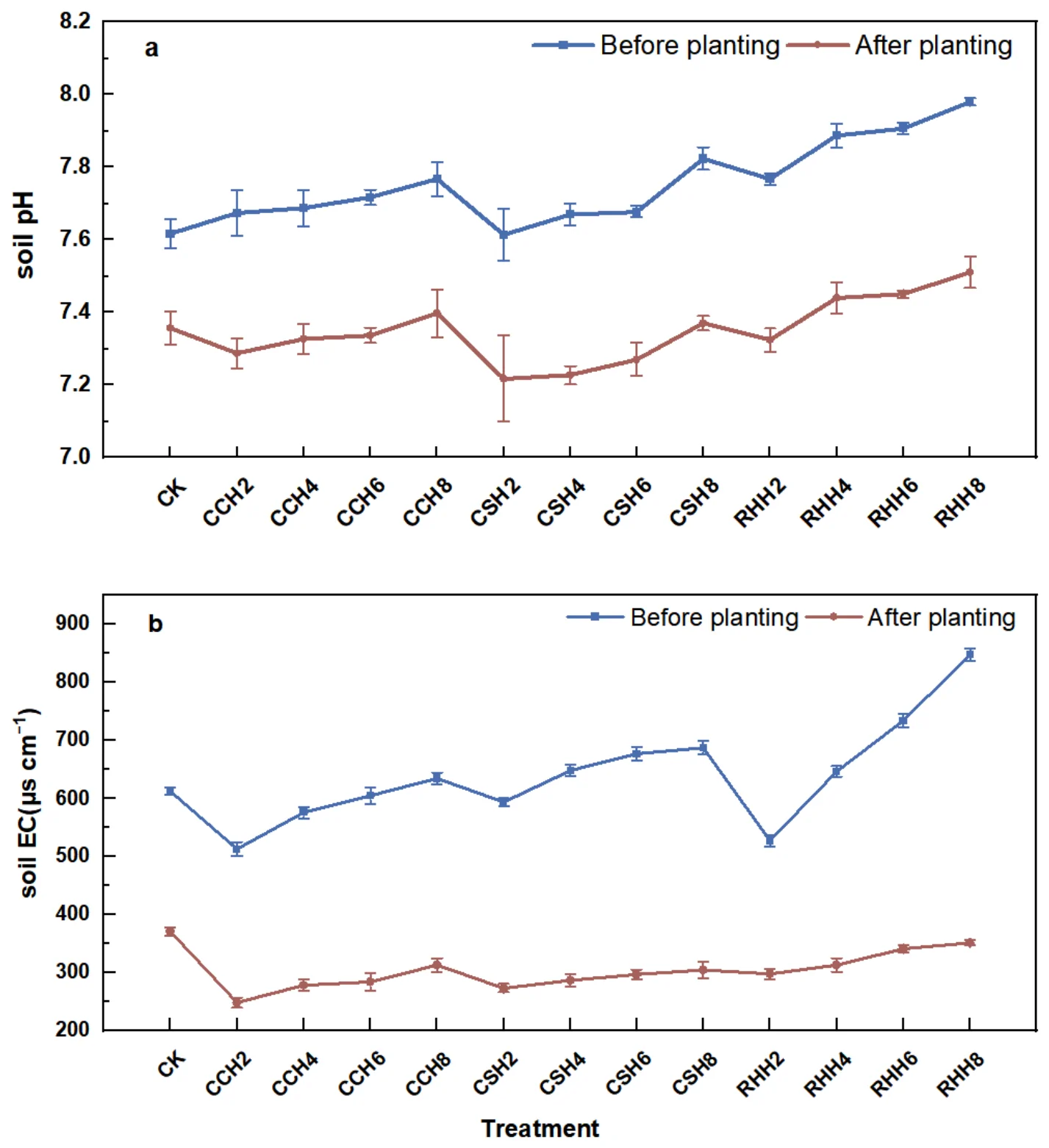
Changes in soil pH and electrical conductivity (EC) before and after ryegrass cultivation under different hydrochar treatments: (**a**) Soil pH and (**b**) soil EC before and after planting. Values are presented as mean ± standard deviation (SD) (*n* = 3 independent pots). CK, control without hydrochar amendment; CCH, corncob hydrochar; CSH, corn straw hydrochar; RHH, rice husk hydrochar; numbers following the abbreviations indicate hydrochar application rates (%).

Two-way mixed-design repeated-measures ANOVA revealed significant treatment × time interactions for both soil pH [F(12, 26) = 4.38, *p* < 0.001, partial η^2^ = 0.669] and EC [F(12, 26) = 284.31, *p* < 0.001, partial η^2^ = 0.992], indicating treatment-dependent changes following ryegrass cultivation (Figure 1; Table S3). Both pH and EC decreased after planting across all treatments. The treatment-specific decrease in pH (ΔpH) differed significantly among treatments [F(12, 26) = 4.384, *p* < 0.001], ranging from 0.260 ± 0.040 in CK to 0.470 ± 0.035 in RHH8 (Table S2). ΔEC also differed significantly among treatments (Welch’s ANOVA, *p* < 0.001), with RHH8 showing the largest decrease (495.9 ± 10.8 μS cm^−1^), compared with 242.0 ± 12.8 μS cm^−1^ in CK (Table S2).

**Figure 2:**
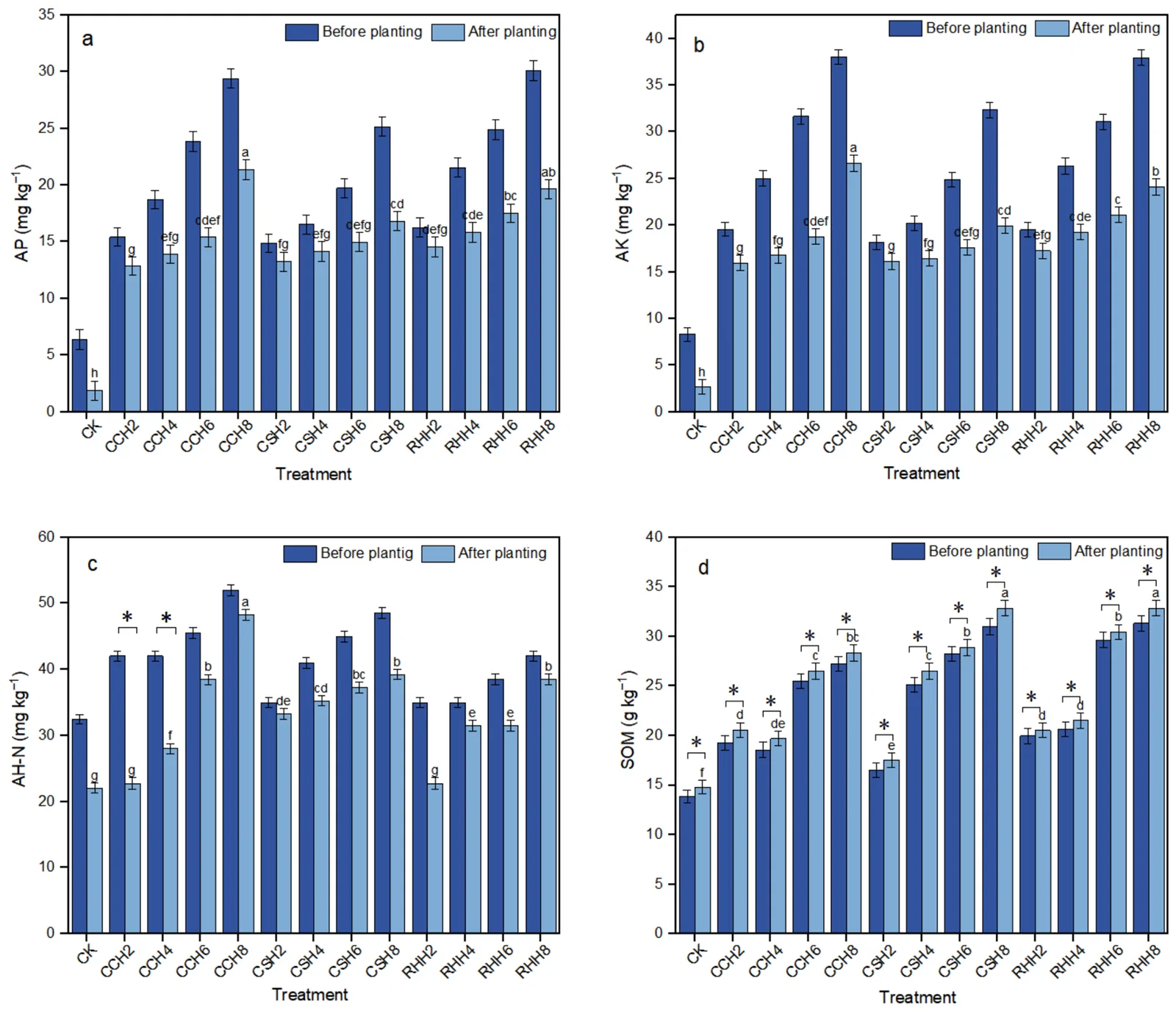
Soil nutrient contents before and after ryegrass cultivation under different hydrochar treatments: (**a**) Available phosphorus (AP), (**b**) available potassium (AK), (**c**) alkali-hydrolyzable nitrogen (AH-N), and (**d**) soil organic matter (SOM). Values are presented as mean ± SD (*n* = 3 independent pots per treatment). Different lowercase letters above the post-planting bars indicate significant differences among treatments; bars sharing at least one letter are not significantly different. Asterisks indicate significant pre- versus post-planting differences within the same treatment; no asterisk indicates a nonsignificant difference. CK, control without hydrochar amendment; CCH, corncob hydrochar; CSH, corn straw hydrochar; RHH, rice husk hydrochar; numbers following the abbreviations indicate hydrochar application rates (%).

Significant treatment × time interactions were detected for AP [F(12, 26) = 9.07, *p* < 0.001, partial η^2^ = 0.807], AK [F(12, 26) = 19.14, *p* < 0.001, partial η^2^ = 0.898], AH-N [F(12, 26) = 29.00, *p* < 0.001, partial η^2^ = 0.930], and SOM [F(12, 26) = 226.89, *p* < 0.001, partial η^2^ = 0.991] (Figure 2). Post-planting treatment effects were also significant for all four variables (all *p* < 0.001, partial η^2^ = 0.976–0.992). Compared with post-planting CK, RHH8 had significantly higher AP, AK, AH-N, and SOM, corresponding to increases of 946.9%, 794.6%, 74.7%, and 121.8%, respectively (Table S4). Within-treatment comparisons showed no significant pre- to post-planting changes in AP or AK after Holm adjustment; AH-N decreased significantly only in CCH2 and CCH4, whereas SOM increased significantly in all treatments (*p*_Holm < 0.05; Table S5).

#### 2.1.2. Ryegrass Growth and Root Responses

**Figure 3:**
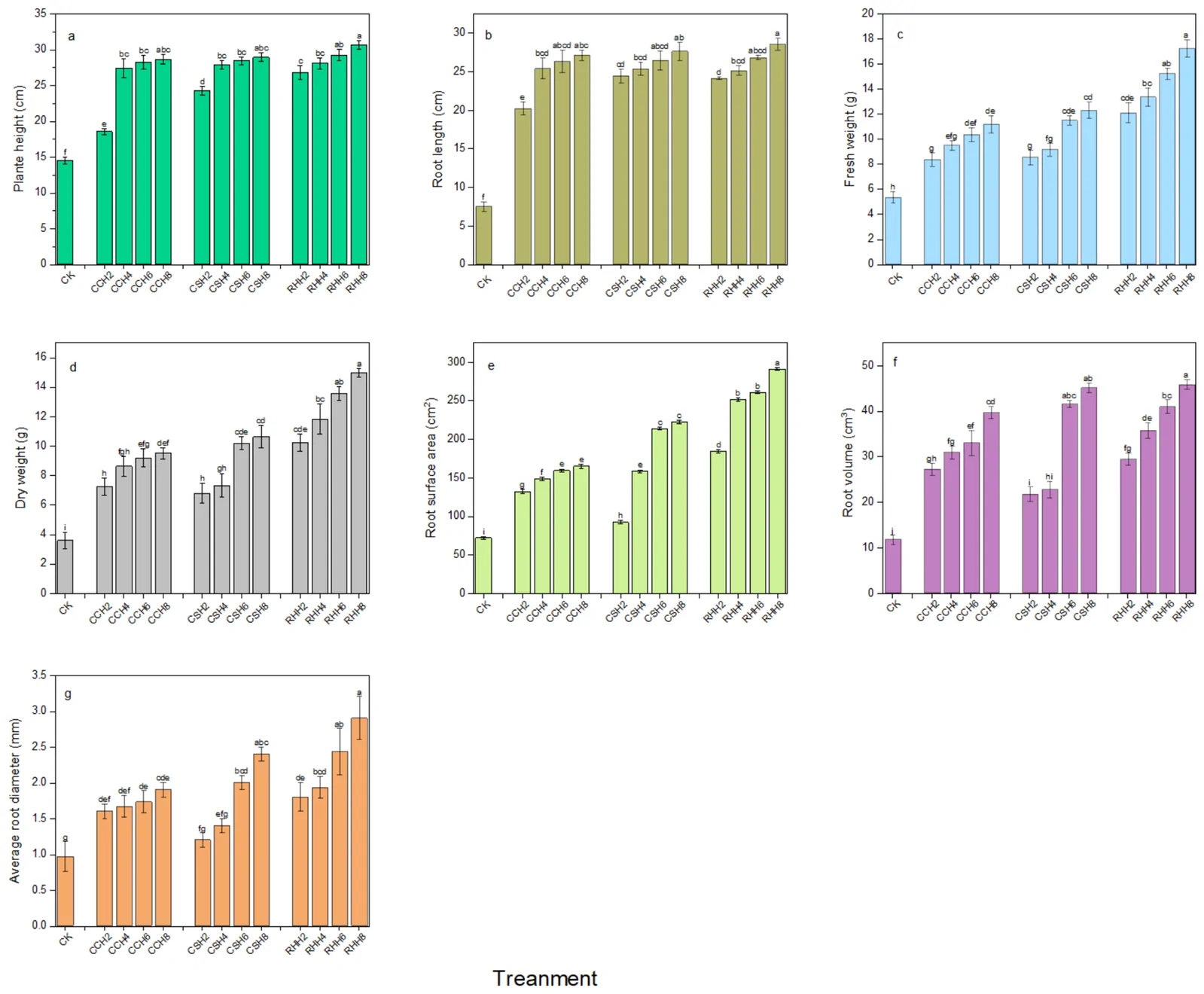
Effects of hydrochar amendment on ryegrass growth and root traits: (**a**) plant height; (**b**) root length; (**c**) fresh weight; (**d**) dry weight; (**e**) root surface area; (**f**) root volume; and (**g**) average root diameter. Values are presented as mean ± SD (*n* = 3 independent pots per treatment), with each pot treated as the independent experimental unit. Different lowercase letters indicate significant differences among treatments; treatments sharing at least one letter are not significantly different. CK, control without hydrochar amendment; CCH, corncob hydrochar; CSH, corn straw hydrochar; RHH, rice husk hydrochar; numbers following the abbreviations indicate hydrochar application rates (%).

Hydrochar amendment significantly affected all measured ryegrass growth and root traits, including plant height, root length, fresh and dry weights, root surface area, root volume, and average root diameter (all *p* < 0.001; η^2^ = 0.924–0.999; Figure 3; Table S6). RHH8 showed the highest mean values for all seven traits (Table S7), with increases of 111.0–315.7% relative to CK. The corresponding RHH8–CK mean differences were 1.93–218.87 in their respective measurement units, with all 95% CIs excluding zero (Table S6).

### 2.2. Effects of Hydrochar and AMF on Sandy Soil Improvement

#### 2.2.1. AMF Root Colonization

**Figure 4:**
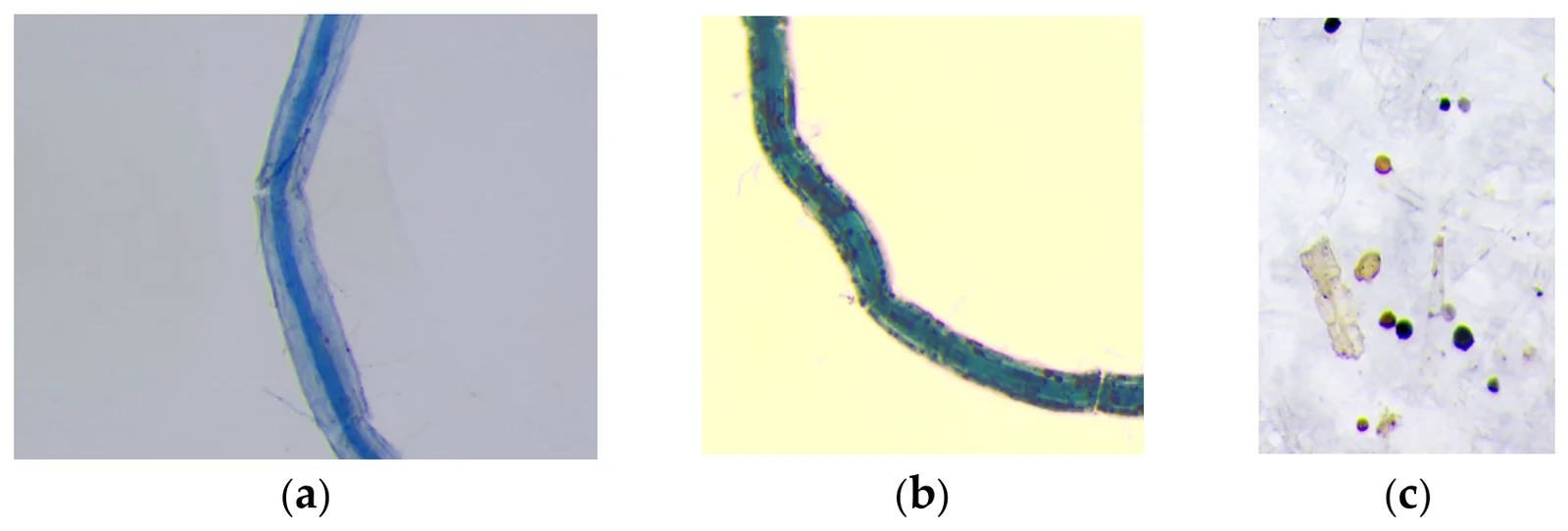
Microscopic observations of AMF root colonization and spores: (**a**) non-colonized root from the non-inoculated S-RHH treatment; (**b**) AMF-colonized root from S-RHH-AMF; (**c**) AMF spores observed in the rhizosphere soil of S-RHH-AMF.

No AMF colonization was detected in any of the four non-inoculated treatments (CK, S, S-CSH, and S-RHH; colonization frequency = 0% in all cases), whereas typical AMF structures were observed in S-RHH-AMF (Figure 4). Root colonization frequency in S-RHH-AMF reached 40.76 ± 2.69%, and rhizosphere spore abundance was 466 ± 13.53 spores 5 g^−1^ soil (mean ± SD, *n* = 3), confirming successful AMF colonization under the experimental conditions.

#### 2.2.2. Rhizobox Soil Responses

**Figure 5:**
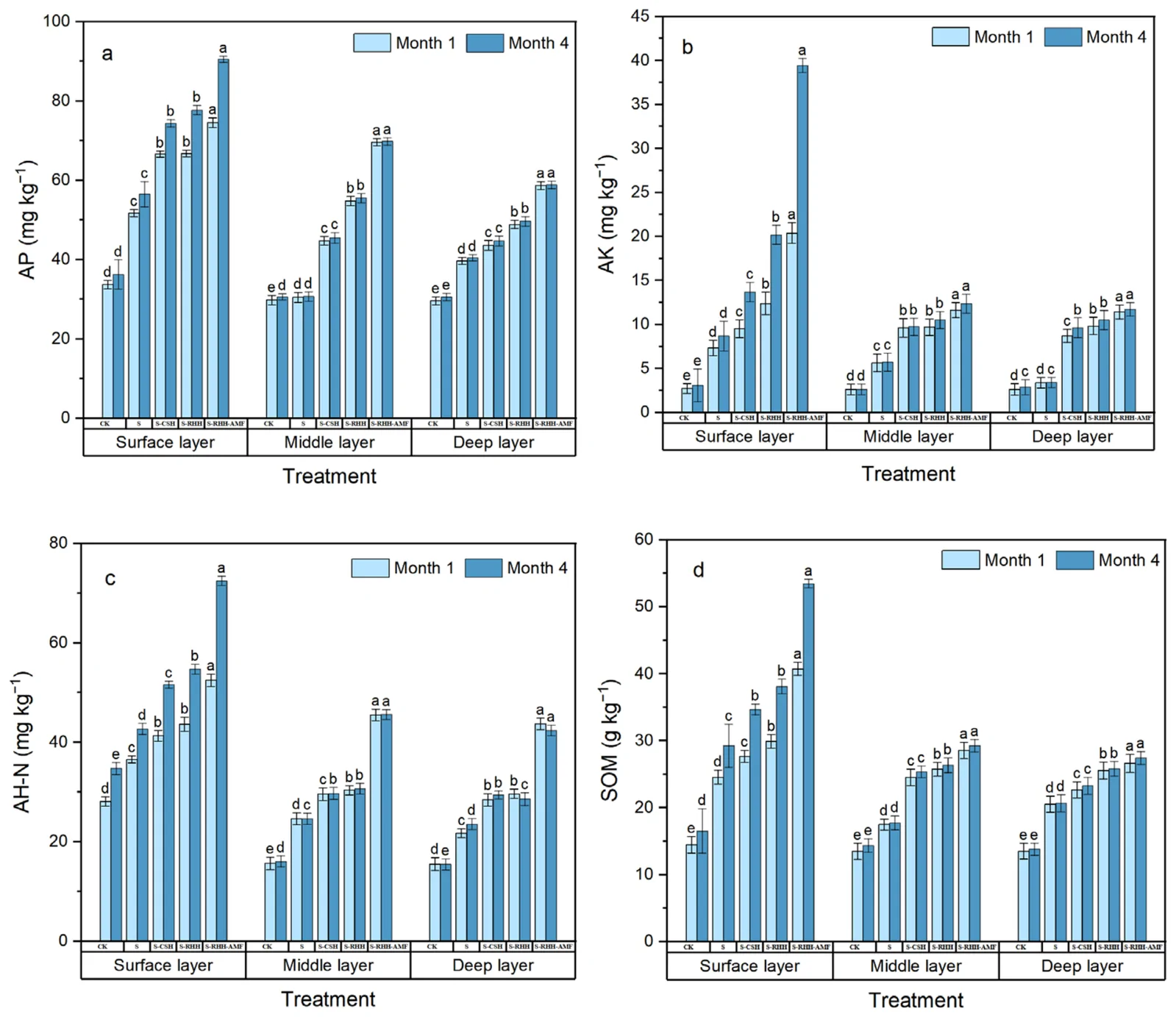
Soil nutrient contents at different soil depths in the rhizobox experiment at Month 1 and Month 4 after planting: (**a**) available phosphorus (AP), (**b**) available potassium (AK), (**c**) alkali-hydrolyzable nitrogen (AH-N), and (**d**) soil organic matter (SOM). Values are presented as mean ± SD (*n* = 3 treatment compartments per treatment, one in each rhizobox). Different lowercase letters indicate significant differences among treatments within the same sampling time, soil layer, and nutrient; treatments sharing at least one letter are not significantly different (*p* < 0.05). CK, unamended sandy soil control; S, coal gangue-amended sandy soil; S-CSH, S amended with corn straw hydrochar; S-RHH, S amended with rice husk hydrochar; S-RHH-AMF, S-RHH inoculated with arbuscular mycorrhizal fungi.

Blocked repeated-measures analyses showed that soil AH-N, AP, AK, and SOM were significantly affected by treatment, soil layer, sampling time, and all associated interactions (all *p* < 0.001; Table S8). At Month 4, S-RHH-AMF showed the highest estimated marginal means (EMMs) for all four nutrients in the 0–10 cm layer (Table S9), consistent with the treatment pattern shown in Figure 5. Compared with CK at the Month 4 endpoint, S-RHH-AMF increased AH-N, AP, AK, and SOM by 37.73, 54.05, 36.39, and 36.95, respectively, with all 95% CIs excluding zero (Tukey-adjusted *p* < 0.001; Table S10).

**Figure 6:**
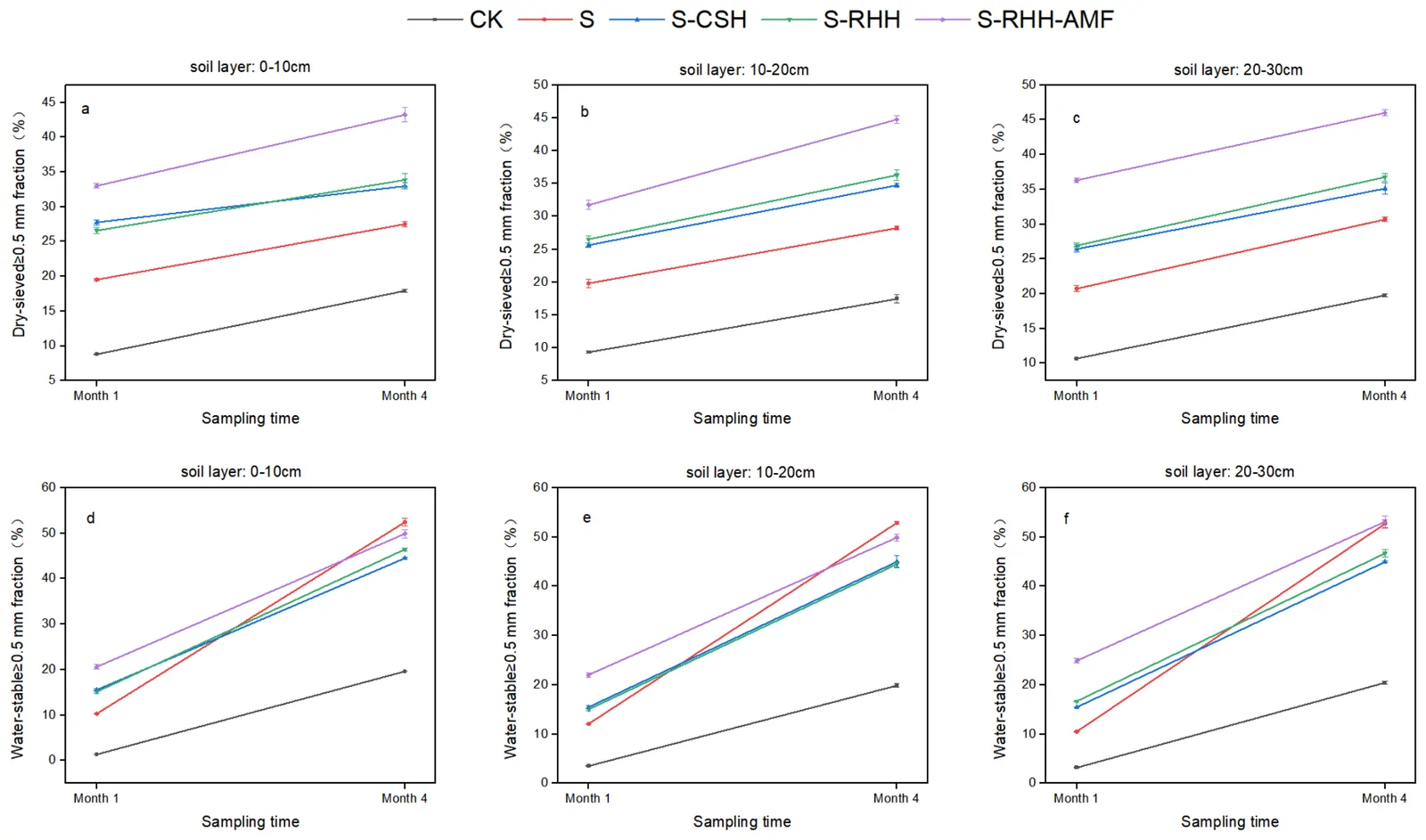
Temporal changes in soil macroaggregate fractions under different treatments across soil layers in the rhizobox experiment. Dry-sieved ≥0.5 mm aggregate fractions in the (**a**) 0–10, (**b**) 10–20, and (**c**) 20–30 cm soil layers, and water-stable ≥0.5 mm aggregate fractions in the (**d**) 0–10, (**e**) 10–20, and (**f**) 20–30 cm soil layers were determined at Month 1 and Month 4. Values are presented as mean ± SD (*n* = 3 treatment compartments per treatment, one in each rhizobox). CK, unamended sandy soil control; S, coal gangue-amended sandy soil; S-CSH, S amended with corn straw hydrochar; S-RHH, S amended with rice husk hydrochar; S-RHH-AMF, S-RHH inoculated with arbuscular mycorrhizal fungi.

Blocked repeated-measures analyses showed pronounced treatment-, soil-layer-, and time-dependent responses in soil aggregation (Figure 6; Tables S11–S14). For the dry-sieved ≥0.5 mm fraction, treatment, soil layer, time, and all interaction terms were significant (*p* < 0.001), including a significant treatment × soil layer × time interaction (F(8, 20) = 9.60, partial η^2^ = 0.793; Table S11). Accordingly, treatment effects were significant within each sampling time × soil layer combination (all *p* < 0.001; Table S12), and the temporal changes differed among treatments and soil layers (Figure 6a–c). By Month 4, the dry-sieved ≥0.5 mm fraction had increased relative to Month 1 across treatments, with S-RHH-AMF consistently showing high values across the three soil layers. At the Month 4 0–10 cm endpoint, S-RHH-AMF had the highest estimated marginal mean (43.22%), compared with 17.92% under CK (Tukey-adjusted *p* < 0.001; Tables S13 and S14). For the water-stable ≥0.5 mm fraction, treatment, soil layer, time, and all two-way interactions were significant (*p* < 0.001), whereas the treatment × soil layer × time interaction was not significant (F(8, 20) = 1.83, *p* = 0.131, partial η^2^ = 0.422; Table S11). Nevertheless, significant treatment effects occurred within all individual sampling time × soil layer combinations (all *p* < 0.001; Table S12), with marked increases from Month 1 to Month 4 across the soil profile (Figure 6d–f). At the Month 4 0–10 cm endpoint, the water-stable ≥0.5 mm fraction was highest under S (52.44%), followed by S-RHH-AMF (49.90%); these two treatments differed significantly (Tukey-adjusted *p* = 0.0018; Tables S13 and S14). Thus, the treatment pattern differed between dry-sieved macroaggregate formation and water-stable aggregation.

**Figure 7:**
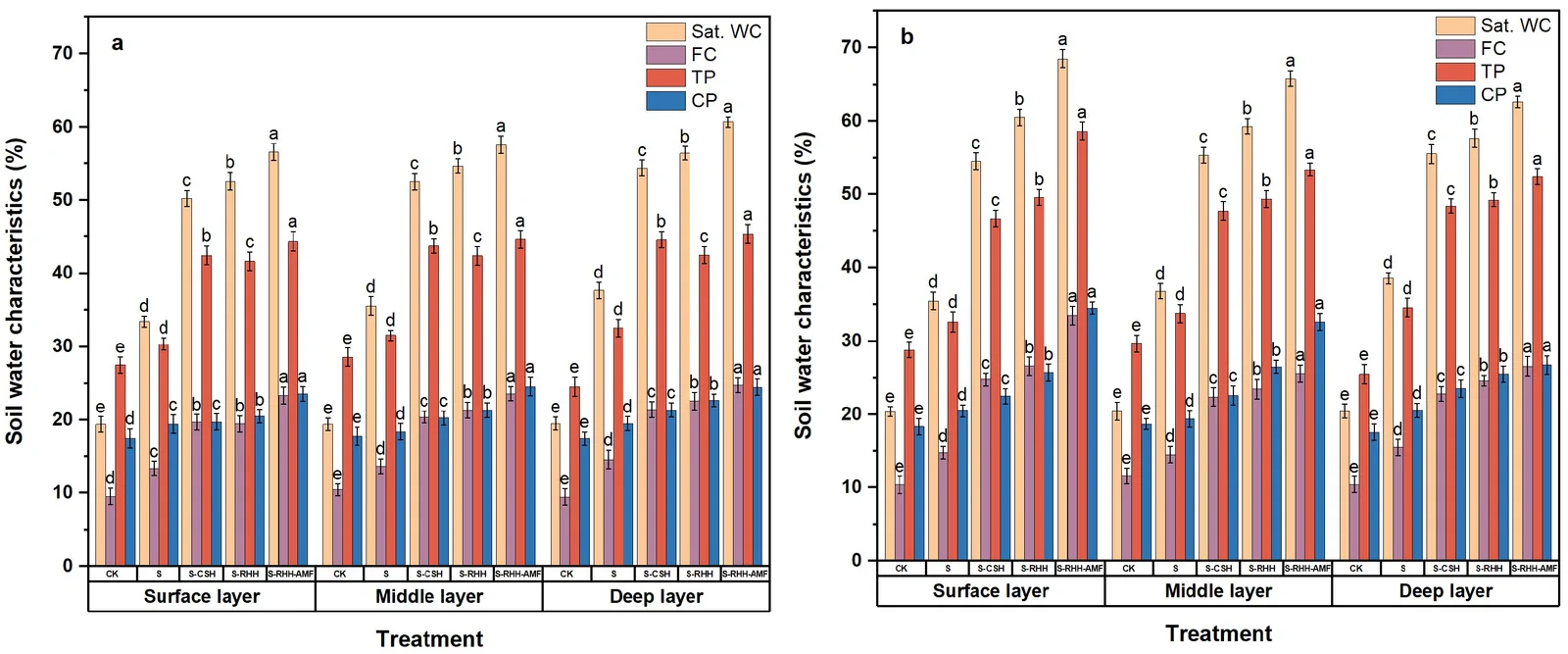
Rhizobox soil water characteristics under different treatments across soil layers at Month 1 and Month 4 after planting: (**a**) Month 1 and (**b**) Month 4. Saturated water content (Sat.WC), field capacity (FC), total porosity (TP), and capillary porosity (CP) were measured in the 0–10, 10–20, and 20–30 cm soil layers. Values are presented as mean ± SD (*n* = 3 treatment compartments per treatment, one in each rhizobox). Different lowercase letters indicate significant differences among treatments within the same sampling time, soil layer, and water characteristic; treatments sharing at least one letter are not significantly different (*p* < 0.05). CK, unamended sandy soil control; S, coal gangue-amended sandy soil; S-CSH, S amended with corn straw hydrochar; S-RHH, S amended with rice husk hydrochar; S-RHH-AMF, S-RHH inoculated with arbuscular mycorrhizal fungi.

Blocked repeated-measures analyses showed that soil water characteristics were significantly affected by treatment, soil layer, sampling time, and their interactions (all *p* < 0.001; Table S15). Significant treatment × soil layer × sampling time interactions were observed for saturated water content (Sat.WC), field capacity (FC), total porosity (TP), and capillary porosity (CP) (partial η^2^ = 0.973–0.986), with significant treatment effects at every sampling time × soil layer combination (Table S16; Figure 7). At Month 4, S-RHH-AMF produced the highest EMMs for all four properties in the 0–10 cm layer (Table S17), exceeding CK by 48.11, 23.12, 29.83, and 16.20 percentage points for Sat.WC, FC, TP, and CP, respectively (all Tukey-adjusted *p* < 0.001; Table S18).

#### 2.2.3. Alfalfa Maximum Root Length

**Figure 8:**
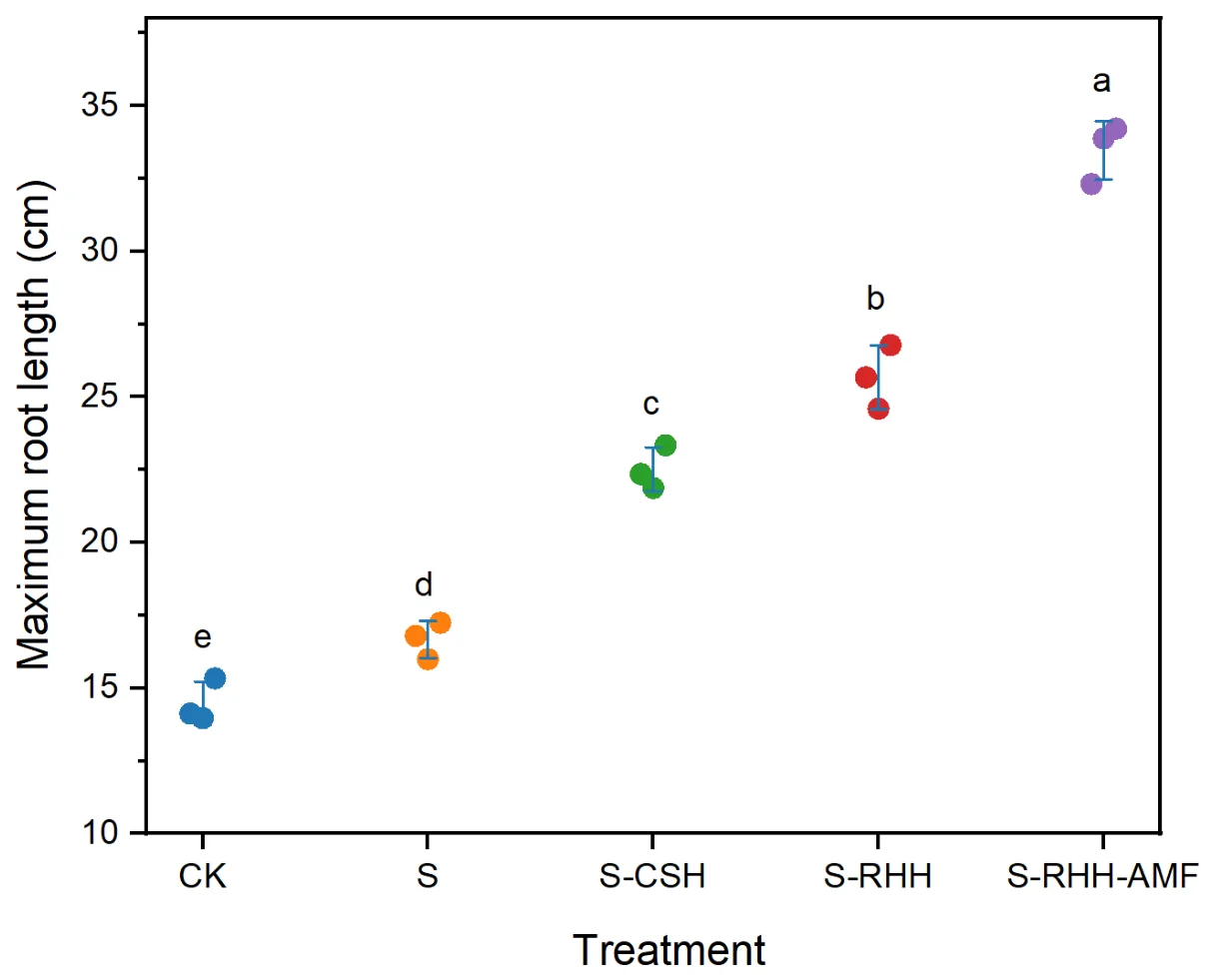
Maximum root length of alfalfa under different treatments at the end of the four-month rhizobox experiment. Points represent individual observations from treatment compartments; error bars indicate mean ± SD (*n* = 3 treatment compartments per treatment, one in each rhizobox). Different lowercase letters indicate significant differences among treatments; treatments sharing at least one letter are not significantly different (*p* < 0.05). CK, unamended sandy soil control; S, coal gangue-amended sandy soil; S-CSH, S amended with corn straw hydrochar; S-RHH, S amended with rice husk hydrochar; S-RHH-AMF, S-RHH inoculated with arbuscular mycorrhizal fungi.

A blocked analysis showed that treatment significantly affected alfalfa maximum root length at the end of the four-month rhizobox experiment [F(4, 8) = 576.44, *p* < 0.001, partial η^2^ = 0.997; Figure 8; Supplementary Table S19]. Maximum root length increased from CK to S, S-CSH, S-RHH, and S-RHH-AMF, with significant differences among all treatments (Supplementary Table S20). S-RHH-AMF showed the greatest maximum root length (33.46 cm), exceeding CK by 18.99 cm (95% CI: 17.45–20.53 cm, Tukey-adjusted *p* < 0.001).

### 2.3. Heavy Metal Concentrations in Soils and Plants

#### 2.3.1. Total Heavy Metal Concentrations in the Initial Materials and Experimental Soils

The total concentrations of As, Cd, Cr, Cu, Hg, Ni, Pb, and Zn in the initial soil and amendment materials were determined to characterize their heavy metal background levels and provide a baseline for interpreting subsequent changes in soil and plant heavy metal concentrations (Table 1).

In the pot experiment, total concentrations of As, Cd, Cr, Cu, Hg, Ni, Pb, and Zn differed significantly among treatments (all *p* ≤ 0.002; Tables S21 and S22). Application rate significantly affected all eight elements, whereas hydrochar type significantly affected all elements except Cd; a significant type × rate interaction was detected only for Ni (Tables S23 and S24). Compared with CK, several hydrochar treatments significantly altered individual metal concentrations, with the complete treatment means and Dunnett-adjusted comparisons provided in Table S21.

In the rhizobox experiment, repeated-measures analyses accounting for rhizobox blocking showed significant treatment effects on all eight soil heavy metals (all *p* < 0.001), whereas interactions between treatment and soil layer were not significant (Table S25). Significant effects of soil layers were detected only for Cr and Zn, and comparisons among soil layers are presented in Table S28. Tables S26 and S27 report the estimated treatment means and treatment comparisons averaged across the three soil layers.

The total concentrations of As, Cd, Cr, Cu, Hg, Ni, Pb, and Zn in both pot and rhizobox soils were below the corresponding risk screening values of GB 15618-2018 for agricultural land (Table 2).

#### 2.3.2. Heavy Metal Concentrations in Plant Tissues

In the pot experiment, heavy metal concentrations in ryegrass were significantly affected by treatment and plant organ (all *p* < 0.001; Tables S29 and S30). Significant treatment × organ interactions were detected for As, Cd, Cr, Cu, Ni, Pb, and Zn, whereas the interaction was not significant for Hg (*p* = 0.889; Table S30). All hydrochar treatments significantly reduced As, Cd, Cr, and Hg concentrations in both shoots and roots relative to CK, whereas the responses of Cu, Ni, Pb, and Zn were more treatment- and organ-dependent (Table S31). Detailed organ-specific comparisons and the effects of hydrochar type and application rate are provided in Table S32.

In the rhizobox experiment, repeated-measures analyses accounting for rhizobox blocking showed significant treatment effects for all eight metals. Significant interactions between treatment and plant organ were detected for As, Cd, Cr, Cu, Ni, Pb, and Zn, but not for Hg (Table S33). Planned comparisons showed that, relative to S, S-CSH, S-RHH, and S-RHH-AMF significantly reduced As, Cr, Cu, Ni, Pb, and Zn in both shoots and roots; Cd was also significantly reduced in roots under S-RHH (Table S34). Compared with S-RHH, S-RHH-AMF further reduced Cu and Zn in shoots and Cr, Cu, Ni, Pb, and Zn in roots (Table S34).

## 3. Discussion

### 3.1. Hydrochar Effects on Soil Properties and Ryegrass Growth

Ryegrass cultivation decreased soil pH and EC across treatments, including CK, indicating that plant growth contributed to the temporal changes in soil chemistry. Nutrient uptake and rhizosphere processes can alter proton balance and soluble ion concentrations during plant growth [34]. Hydrochar modified the magnitude of these changes, with stronger responses generally occurring at higher application rates. Rather than reflecting a simple liming effect, the pH and EC patterns likely resulted from the combined influence of plant growth and hydrochar on the chemical environment of the amended sandy soil. Similar variability in soil responses to hydrochar has been reported across different soil and amendment conditions [17].

Hydrochar also improved soil nutrient status and SOM, accompanied by better ryegrass growth. This correspondence suggests that improved soil fertility supported plant establishment in the coarse-textured substrate. The differences among hydrochars were consistent with their physicochemical characteristics. In particular, RHH combined a larger specific surface area with higher organic carbon and nutrient contents, which may favor nutrient retention and supply and help explain its stronger overall soil and plant responses. Previous studies have similarly reported improvements in soil fertility following hydrochar application, although plant responses vary considerably with hydrochar properties and soil conditions [17,18]. Fermented chicken manure provided a common nutrient input across treatments, while the differences among treatments indicate that hydrochar type and application rate further influenced nutrient dynamics and plant performance.

The stronger ryegrass response to RHH further supports the importance of evaluating soil and plant responses together when selecting hydrochar for restoration. Improved soil fertility alone does not necessarily translate into greater plant growth, as hydrochar can produce contrasting plant responses even when some soil properties improve [12,17]. In the present study, the combined soil and ryegrass responses therefore provided a practical basis for selecting CSH and RHH for further evaluation. The 8% rate was carried forward because it showed the strongest overall performance within the tested range, rather than as an assumed optimum. The subsequent rhizobox experiment was designed to examine whether these improvements were maintained in a vertically structured soil environment.

### 3.2. Soil and Alfalfa Responses in the Rhizobox Experiment

The rhizobox experiment showed that the benefits of hydrochar extended beyond nutrient improvement to changes in soil structure and water retention. CSH and RHH maintained higher nutrient levels across the soil profile, with RHH generally showing the stronger response. This agrees with the pot experiment and may be related to the greater surface area and nutrient content of RHH. The variation among soil layers and between sampling times further indicates that hydrochar effects developed dynamically within the soil profile rather than remaining confined to the amended surface layer. Such responses are important in sandy substrates, where poor nutrient retention and rapid water loss are major constraints on plant establishment [17].

Improvements in aggregation and water retention provide a physical basis for the better performance of the amended soil. Hydrochar can introduce additional pore space and promote the formation of larger aggregates, thereby modifying water storage and pore connectivity in coarse-textured soils [12]. AMF inoculation further enhanced several soil properties in the RHH treatment. Established AMF colonization can contribute to aggregate stabilization through hyphal networks and associated organic compounds while also extending nutrient acquisition beyond the root depletion zone [35]. These biological processes may complement the physical and chemical effects of hydrochar, helping to maintain a soil environment more favorable for nutrient and water retention. The treatment-dependent differences between dry-sieved and water-stable aggregates also suggest that aggregate abundance and resistance to water disruption represent distinct aspects of structural improvement.

These changes were accompanied by greater alfalfa root extension, with the strongest response occurring under RHH combined with AMF. A more favorable combination of nutrient availability, porosity, and water retention can reduce constraints on root penetration and support root exploration in sandy substrates [12,35]. Deeper rooting is particularly relevant to vegetation establishment because it increases the soil volume accessible for water and nutrient acquisition. The pot and rhizobox experiments therefore provide complementary evidence: the former identified hydrochars with stronger soil and ryegrass responses, whereas the latter showed that the selected amendments also improved the vertical soil environment and supported greater alfalfa root extension. Together, these results indicate that RHH combined with AMF has particular potential for improving the functional quality of coal gangue-amended sandy soil.

### 3.3. Heavy Metal Responses in Soil and Plants

Hydrochar altered heavy metal behavior in the soil–plant system, with the clearest responses occurring in plant accumulation and partitioning. The physicochemical characteristics of the hydrochars provide a basis for these effects. Their porous carbon matrices and surface functional groups provide reactive interfaces for metal interactions, while mineral components can further influence metal retention [36,37]. In particular, RHH combined the highest specific surface area and organic carbon content with relatively high SiO_2_ and P_2_O_5_ contents. These carbonaceous and mineral characteristics may help explain its favorable performance, as hydrochar–metal interactions are strongly governed by surface properties, mineral composition, and the chemistry of individual metals [36,37].

Plant responses were correspondingly element-dependent. Hydrochar consistently reduced As, Cd, Cr, and Hg accumulation in ryegrass, whereas Cu, Ni, Pb, and Zn varied more among treatments and plant organs, consistent with the dependence of plant metal uptake on amendment properties, soil conditions, and metal chemistry [36,38]. In the rhizobox experiment, CSH and RHH reduced the concentrations of several metals in alfalfa tissues, while AMF addition to RHH produced further reductions for selected metals, particularly in roots. AMF can modify metal partitioning at the soil–root interface through fungal and root-associated retention and changes in rhizosphere processes [39,40]. Together with the improved soil structure and greater root extension observed under RHH-AMF, these responses were associated with changes in metal partitioning within the amended soil–plant system. Meanwhile, total soil concentrations of all measured metals remained below the referenced risk screening values during the experimental period.

### 3.4. Implications, Limitations, and Future Research

**Figure 9:**
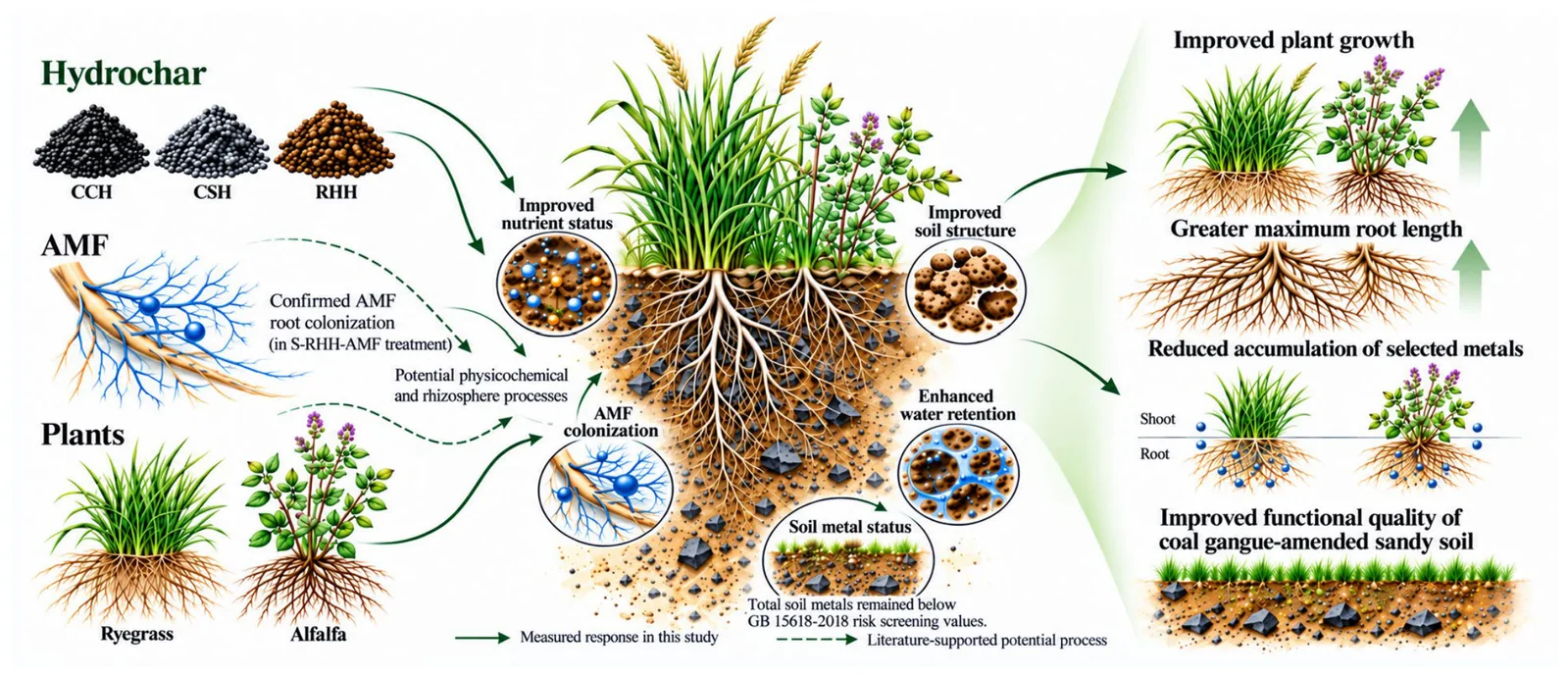
Conceptual framework for restoration of coal gangue-amended sandy soil using hydrochar and AMF. Solid arrows indicate responses measured in this study, whereas dashed arrows represent potential processes supported by previous studies. CCH, corncob hydrochar; CSH, corn straw hydrochar; RHH, rice husk hydrochar; AMF, arbuscular mycorrhizal fungi.

Restoration of coal gangue-amended sandy soil requires simultaneous improvement of soil fertility, physical functioning, and plant establishment rather than modification of any single soil property. From this perspective, the present findings provide an integrated framework linking hydrochar amendment with plant and microbial processes (Figure 9). Hydrochar provides physicochemical attributes relevant to nutrient retention, soil structure, water availability, and metal behavior, whereas roots and AMF introduce biological processes that may further shape the developing soil environment [17,35,41]. Figure 9 therefore summarizes the relationships supported by the experiments while distinguishing them from potential processes inferred from hydrochar characteristics and previous studies. An overview of the major soil and plant responses and their statistical support across treatments is provided in Table S35.

The practical value of this framework lies in evaluating restoration performance across several functions that are simultaneously limiting in coarse-textured substrates. Combining hydrochar with vegetation establishment also provides a route for coupling biomass utilization with the reuse of coal gangue, while AMF represents a potential biological complement to material amendment. This integrated perspective extends beyond the conventional evaluation of hydrochar primarily through soil chemical improvement and emphasizes whether changes in the substrate ultimately support plant establishment and root extension [17,35]. Within the tested range, RHH at 8% produced the strongest overall response, but the present results do not establish 8% as the optimal field rate. Field application will require balancing restoration performance with hydrochar demand, production and transport costs, and application feasibility. Rate optimization under field conditions is therefore necessary before large-scale implementation [15,17].

The interpretation of this framework should nevertheless remain within the experimental scope. The pot and rhizobox experiments represent complementary stages rather than directly comparable systems because they differed in plant species, spatial configuration, and experimental duration. In addition, the fixed, non-randomized treatment positions across rhizoboxes may have confounded treatment effects with compartment-position effects; thus, the observed between-treatment differences should be interpreted cautiously. Future experiments should randomize or counterbalance treatment positions across replicate rhizoboxes. Likewise, the additional responses observed after AMF inoculation were evaluated only in the RHH treatment; without an AMF-only treatment and a complete hydrochar × AMF factorial design, they should not be generalized as a synergistic effect. The metal results provide evidence for changes in plant accumulation and within-plant partitioning, but total soil concentrations cannot resolve changes in metal bioavailability, speciation, or mobility. More broadly, the surface interactions, hyphal effects, and rhizosphere processes represented in Figure 9 remain plausible explanations rather than directly quantified mechanisms [35,41].

These considerations define the next steps toward field application. Multi-season field trials are needed to determine whether improvements in fertility, soil structure, water retention, and vegetation establishment persist under natural climatic and hydrological variability and to identify effective rates at realistic application costs. Such trials should incorporate measurements of metal bioavailability and leaching to evaluate environmental behavior beyond total concentrations. A more complete factorial design would help separate hydrochar and AMF contributions, while measurements of extraradical hyphae, glomalin-related soil proteins, root exudates, microbial communities, and changes in hydrochar surface chemistry during aging would provide direct evidence for the biological and physicochemical processes proposed in Figure 9 [35,41]. Linking these mechanistic measurements with long-term plant performance and field-scale feasibility will be essential for developing a durable restoration strategy for coal gangue-amended sandy soils.

## 4. Materials and Methods

### 4.1. Test Materials

The coal gangue used in this study was obtained from the waste dump of Shanxi Liulin Coal Mine Co., Ltd. (Luliang, China). The sandy soil was collected from Liulin Town, Liulin County, Luliang City, Shanxi Province, China. Its particle composition was 93.60% sand, 4.64% silt, and 1.76% clay. Coal gangue (<0.5 mm) and sandy soil (<2 mm) were mixed at a ratio of 4:6 (*w/w*) as the base substrate. This substrate formulation was selected based on preliminary optimization experiments conducted by our research group, in which different gangue–sandy soil ratios and gangue particle sizes were compared in terms of their overall water retention, infiltration, and evaporation characteristics. The basic physicochemical properties of the sandy soil, coal gangue, and mixed substrate are shown in Table 3.

**Figure 10:**
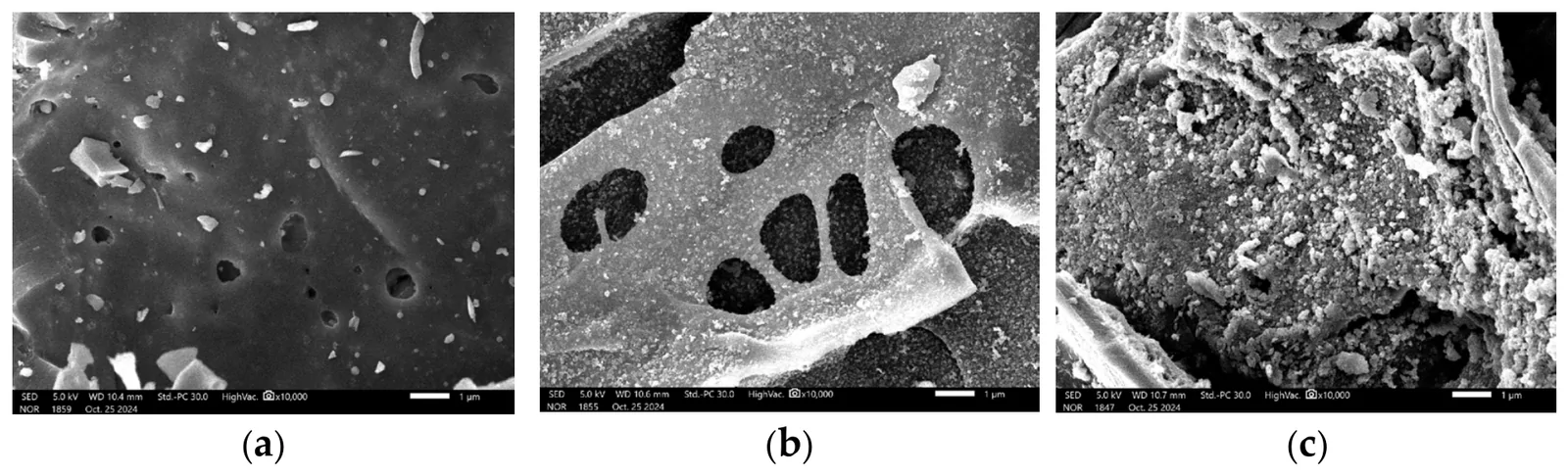
Scanning electron microscopy (SEM) images of the three hydrochars: (**a**) corncob hydrochar (CCH), (**b**) corn straw hydrochar (CSH), and (**c**) rice husk hydrochar (RHH).

**Figure 11:**
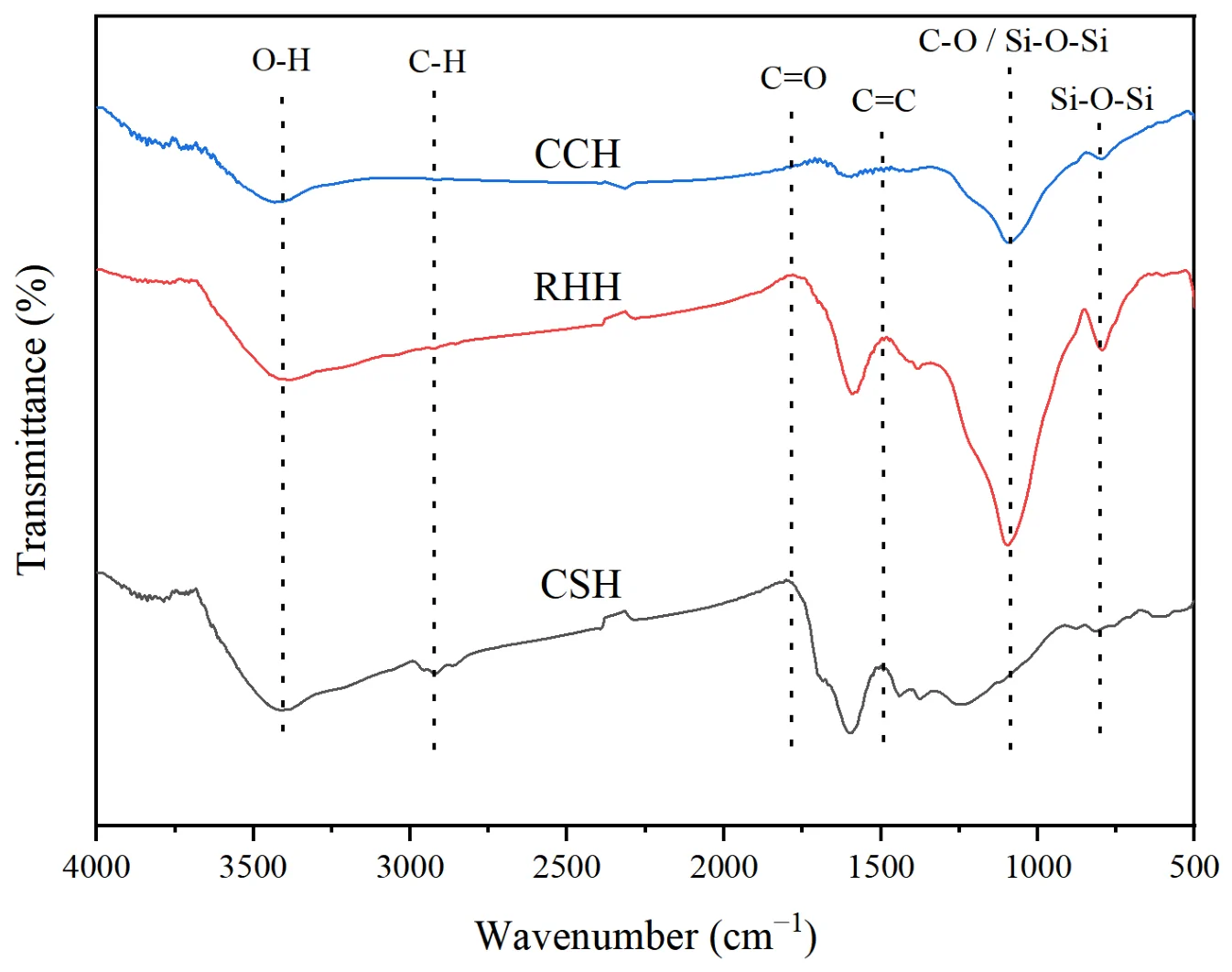
Fourier-transform infrared (FTIR) spectra of the three hydrochars. CCH, corncob hydrochar; CSH, corn straw hydrochar; RHH, rice husk hydrochar.

Three commercial hydrochars were used in this study: corncob hydrochar (CCH), corn straw hydrochar (CSH), and rice husk hydrochar (RHH). All three hydrochars were purchased from Henan Lize Environmental Protection Technology Co., Ltd. (Zhengzhou, Henan Province, China). According to the production information provided by the manufacturer, CCH, CSH, and RHH were produced from corncob, corn straw, and rice husk, respectively, via hydrothermal carbonization (HTC) at 180–250 °C and 2–10 MPa. Their basic physicochemical properties are summarized in Table 4, and their oxide compositions determined by X-ray fluorescence (XRF) analysis are presented in Table 5. The surface morphologies and functional groups of the three hydrochars were characterized by scanning electron microscopy (SEM) and Fourier-transform infrared (FTIR) spectroscopy, respectively (Figure 10 and Figure 11). Commercially fermented chicken manure was purchased from Kunning Wang (Liaoning) Agricultural Co., Ltd. (Tieling, China). The manure was fermented by the manufacturer before supply, and its basic physicochemical properties are provided in Table 4. Arbuscular mycorrhizal fungi (AMF) were purchased from Shanxi Gerun Hetai Co., Ltd. (Taiyuan, China).

### 4.2. Experimental Design

#### 4.2.1. Pot Experiment

Ryegrass (*Lolium perenne* L.) was selected as the test plant because of its short growth cycle, tolerance to infertile conditions, rapid root growth, and sensitivity to changes in the soil environment, making it suitable for screening hydrochar treatments. The coal gangue–sandy soil mixture described in Section 4.1 was used as the base substrate. CCH, CSH, and RHH were each applied at 2%, 4%, 6%, and 8% (*w/w*), generating 12 hydrochar treatments, together with a control without hydrochar addition (CK). Fermented chicken manure was applied at 5% (*w/w*) to all treatments, including CK. The total mass of substrate and amendments was maintained at 450 g per pot. A total of 13 treatments were established, with three replicates per treatment.

The mixtures were placed in plastic pots (8.5 cm high, with a bottom diameter of 7 cm) and pre-incubated for 14 days at 25 °C in the dark. During pre-incubation, pots were weighed every three days and watered to restore substrate moisture to field capacity. Fifteen ryegrass seeds were then sown per pot and thinned to 12 seedlings after emergence. Plants were maintained in a controlled-environment greenhouse at day/night temperatures of 25/20 °C, approximately 34% relative humidity, a 14 h light/10 h dark photoperiod, and a photosynthetic photon flux density of approximately 350 μmol m^−2^ s^−1^ during the light period. Pots were watered every three days to restore soil moisture to field capacity. Soil samples were collected immediately before sowing to establish treatment-specific baseline conditions for evaluating subsequent cultivation-induced changes. Soil and plant samples were collected after two months of growth for subsequent analyses.

#### 4.2.2. Multi-Layer Rhizobox Experiment

**Figure 12:**
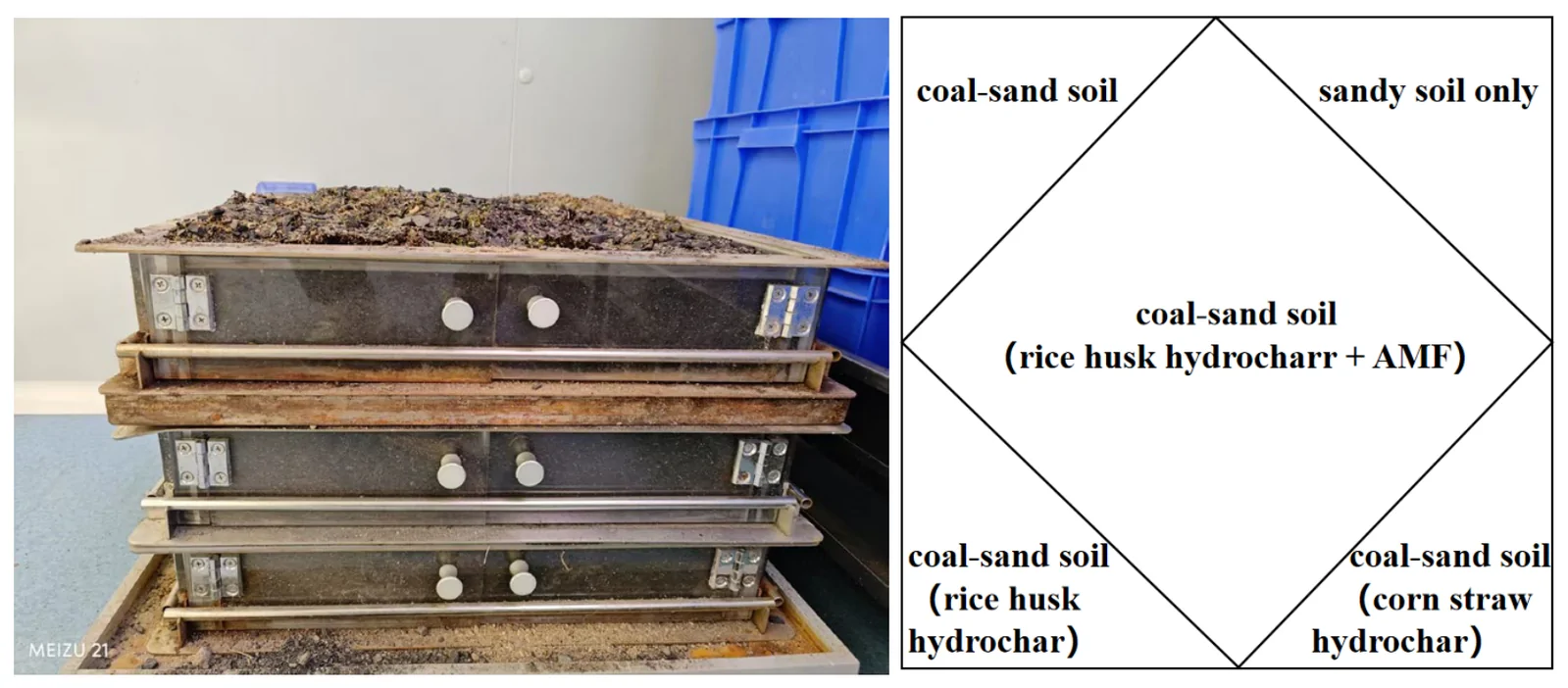
Structural diagram of the multi-layer rhizobox for root visualization.

Considering the overall soil and plant responses in the pot experiment and the physicochemical characteristics of the hydrochars, CSH and RHH were further evaluated at 8%, the best-performing rate among those tested, in a four-month rhizobox experiment. Alfalfa (*Medicago sativa* L.) was selected because its well-developed root system, drought tolerance, and ability to form symbioses with AMF made it suitable for investigating soil responses across depths and the effects of AMF inoculation. Three independent polypropylene rhizoboxes (30 × 30 × 30 cm) were used, each divided into five physically isolated treatment compartments. The 30 cm soil profile was divided into three sampling depth intervals (0–10, 10–20, and 20–30 cm) (Figure 12).

Five treatments were established: CK (sandy soil), S (coal gangue–sandy soil = 4:6, *w/w*), S-CSH (S amended with 8% CSH, *w/w*), S-RHH (S amended with 8% RHH, *w/w*), and S-RHH-AMF (S amended with 8% RHH, *w/w*, and inoculated with AMF). Each treatment was represented once in each of the three rhizoboxes, providing three treatment compartments per treatment (*n* = 3), one in each rhizobox. The treatment compartment was the experimental unit to which the treatment was applied, whereas rhizobox identity served as a blocking factor. Treatment positions were identical among rhizoboxes and were not randomized. All treatments received 5% (*w/w*) fermented chicken manure. For S-RHH-AMF, 5 g of powdered AMF inoculum (≥100 spores g^−1^) was uniformly incorporated into the 0–10 cm soil layer before sowing. Twenty-two alfalfa seeds were sown in each compartment and thinned to 18 plants after emergence. The rhizoboxes were maintained under the same environmental and watering conditions as those used in the pot experiment.

Soil samples were collected at the end of Month 1 and Month 4 after planting from each of the three depth intervals in each treatment compartment. At each depth interval, two 100 cm^3^ ring-core samples were collected, one for aggregate analysis and the other for soil water-related measurements, together with a 10 cm^3^ soil sample for nutrient analysis. At Month 4, a subsample of the 10 cm^3^ soil sample was also used for determination of total heavy metal concentrations. At the end of the experiment, roots were collected to determine the maximum root length in each treatment compartment. Sampling holes created at Month 1 were not refilled. Measurements obtained at different soil depths and sampling times were therefore treated as repeated observations within the same treatment compartment.

### 4.3. Analytical Methods

#### 4.3.1. Hydrochar Characterization

Organic carbon (OC) was determined using a total organic carbon analyzer (Aurora 1030D, OI Analytical, College Station, TX, USA). Total nitrogen (TN) was determined by the Kjeldahl method, available phosphorus (AP) by NaHCO_3_ extraction followed by molybdenum–antimony colorimetry, and total potassium (TK) by inductively coupled plasma optical emission spectrometry (ICP-OES) after digestion. Specific surface area (SSA) and average pore diameter (APD) were determined by N_2_ adsorption–desorption analysis (JW-BK400, Beijing JWGB Instrument Co., Ltd., Beijing, China), with SSA calculated using the Brunauer–Emmett–Teller (BET) method. Surface morphology and functional groups were characterized by scanning electron microscopy (SEM; JSM-IT500HR, JEOL Ltd., Akishima, Tokyo, Japan) and Fourier-transform infrared spectroscopy (FTIR; INVENIO-S, Bruker Optics GmbH & Co. KG, Ettlingen, Germany), respectively. Major oxide composition was determined by X-ray fluorescence spectroscopy (XRF; MEGREZ-α, Beijing Anchor Wisdom Technology Co., Ltd., Beijing, China).

#### 4.3.2. Soil and Plant Analyses

Saturated water content, field capacity, total porosity, and capillary porosity were determined using undisturbed soil cores collected with 100 cm^3^ core rings. The cores were saturated with water for 24 h to obtain the saturated mass, drained on dry sand for 2 h to obtain the drained mass, and subsequently oven-dried at 105 °C to constant weight to obtain the dry mass. The four indices were calculated from these measurements and the known core volume. Soil pH and electrical conductivity (EC) were measured in soil–water suspensions at ratios of 1:2.5 and 1:5 (*w/v*), respectively. Soil organic matter (SOM) was determined by potassium dichromate oxidation with external heating, alkali-hydrolyzable nitrogen (AH-N) by alkaline hydrolysis diffusion, available phosphorus (AP) by NaHCO_3_ extraction followed by molybdenum–antimony colorimetry, and available potassium (AK) by ammonium acetate extraction followed by flame photometry. Water-stable and mechanical aggregates were determined by wet and dry sieving, respectively.

Total heavy metal concentrations in raw materials, soil, and plant samples were determined by inductively coupled plasma optical emission spectrometry (ICP-OES; 5800 VDV, Agilent Technologies, Santa Clara, CA, USA) following microwave digestion (ETHOS UP, Milestone S.p.A., Sorisole, Italy). Ryegrass roots were scanned using a root scanner (MRS-3200A3L, Shanghai Zhongjing Technology Co., Ltd., Shanghai, China), and root surface area, root volume, and average root diameter were quantified using the associated analysis system. AMF colonization was assessed after roots were cleared in 10% KOH at 90 °C for 45 min, acidified in 2% HCl for 5 min, and stained with 0.05% (*w/v*) trypan blue at 90 °C for 30–60 min. For each sample, 20 root segments were examined microscopically, and colonization frequency was calculated as the percentage of colonized segments among those examined. AMF spores were extracted from 5 g of rhizosphere soil by wet sieving followed by sucrose centrifugation and counted microscopically. Colonization was assessed in all treatments, with S-RHH serving as the corresponding non-inoculated treatment for comparison with S-RHH-AMF. Aboveground and belowground plant biomass was determined after oven drying at 60 °C to constant weight. Unless otherwise specified, routine soil chemical analyses followed the procedures described by Bao [42].

### 4.4. Data Analysis

Data are presented as mean ± standard deviation (SD). Pots were treated as independent experimental units, and pot data were analyzed by one-way analysis of variance (ANOVA) or repeated-measures ANOVA as appropriate. In the rhizobox experiment, each treatment compartment was the experimental unit, and rhizobox identity was included as a blocking factor. Treatment was a fixed between-compartment factor, whereas soil depth and sampling time were repeated within-compartment factors when applicable. Variables measured across depth and time were analyzed using blocked repeated-measures ANOVA, with treatment effects evaluated after accounting for rhizobox blocking; significant interactions were examined by simple-effects analyses. Soil heavy metal concentrations were measured only at Month 4 and analyzed with soil depth as the repeated factor. Heavy metal concentrations in alfalfa tissues were analyzed with plant organ as the repeated factor, whereas maximum root length was analyzed by blocked ANOVA. Tukey, Dunnett, or Holm adjustments were applied to post hoc or planned comparisons, as appropriate. Welch’s ANOVA with Games–Howell comparisons was used when variances were unequal. Model assumptions were checked before analysis, and data were transformed when necessary. Ryegrass fresh weight and root surface area were log-transformed; statistical tests were performed on the transformed values, whereas descriptive values are reported on the original scale. Effect sizes and confidence intervals (CIs) are reported where appropriate. Statistical significance was set at *p* < 0.05.

## 5. Conclusions

In a coal gangue-amended sandy soil substrate, 8% rice husk hydrochar (RHH) improved soil fertility, water retention, aggregate stability, and plant performance, with additional improvements in several parameters following AMF inoculation. Hydrochar also reduced the accumulation of several heavy metals in plants, while total soil metal concentrations remained below the referenced risk screening values during the experimental period. These findings demonstrate the potential of RHH, with AMF as a biological complement, for the ecological restoration of coal gangue-amended sandy soils. Long-term field trials are required to optimize application rates and evaluate the persistence and practical feasibility of these effects.

## Figures and Tables

**Table 1:** Total concentrations of heavy metals in the initial soil and amendment materials used in the experiments (mg kg^−1^).

Material	As	Cd	Cr	Cu	Hg	Ni	Pb	Zn
Sandy soil	0.65	0.06	9.84	3.12	0.03	5.67	12.45	18.95
Coal gangue	4.21	0.12	28.65	18.24	0.15	22.51	42.15	85.64
Fermented chicken manure	5.82	0.28	25.34	28.56	0.28	7.12	38.92	152.34
Corncob hydrochar	0.62	0.09	10.24	15.32	0.12	7.15	8.45	39.24
Corn straw hydrochar	0.58	0.08	8.65	12.45	0.15	6.52	7.82	34.51
Rice husk hydrochar	0.48	0.07	6.85	8.24	0.08	4.85	5.62	22.15

Note: Values represent total concentrations of heavy metals determined after microwave digestion by inductively coupled plasma optical emission spectrometry (ICP-OES).

**Table 2:** Ranges of total heavy metal concentrations in pot and rhizobox soils compared with the risk screening values of GB 15618-2018 (mg kg^−1^).

Soil sample	As	Cd	Cr	Cu	Hg	Ni	Pb	Zn
Pot soil (13 treatments)	1.75–2.43	0.08–0.09	12.91–16.14	7.27–9.18	0.06–0.09	9.58–11.93	16.73–23.90	33.89–46.54
Rhizobox soil (0–10 cm, Month 4; 5 treatments)	0.64–2.15	0.06–0.09	10.20–15.77	3.19–8.99	0.03–0.09	5.95–11.78	12.71–23.90	18.93–46.90
GB 15618-2018 risk screening value	25	0.6	250	100	3.4	190	170	300

Note: Values for pot and rhizobox soils are presented as ranges of treatment means. All metal concentrations represent total concentrations. Risk screening values were taken from GB 15618-2018 for agricultural land other than paddy fields at pH > 7.5. All treatment means for the eight metals were below the corresponding risk screening values.

**Table 3:** Basic physicochemical properties of the sandy soil, coal gangue, and their mixed substrate.

Material	pH	EC (μS cm^−1^)	Bulk Density (g cm^−3^)	AH-N (mg kg^−1^)	AP (mg kg^−1^)	AK (mg kg^−1^)	SOM (g kg^−1^)
Sandy soil <2 mm	7.89	144.33	1.43	1.75	9.16	31.00	5.96
Coal gangue <0.5 mm	8.73	739.12	1.31	19.25	22.40	142.33	48.15
Mixed substrate (coal gangue–sandy soil = 4:6)	8.84	359.87	1.28	14.00	33.30	105.00	33.71

Note: EC, electrical conductivity; AH-N, alkali-hydrolyzable nitrogen; AP, available phosphorus; AK, available potassium; SOM, soil organic matter.

**Table 4:** Basic physicochemical properties of the hydrochars and fermented chicken manure used in this study.

Material	pH	SSA (m^2^ g^−1^)	APD (nm)	OC (g kg^−1^)	TN (g kg^−1^)	AP (mg kg^−1^)	TK (g kg^−1^)
Corncob hydrochar	8.47	5.35	6.99	375.80	2.35	2.54	3.86
Corn straw hydrochar	9.46	37.65	16.53	422.10	3.64	2.31	5.22
Rice husk hydrochar	8.14	57.32	3.37	440.60	5.21	6.16	7.32
Fermented chicken manure	7.44	—	—	—	22.36	18.86	—

Note: SSA, specific surface area; APD, average pore diameter; OC, organic carbon; TN, total nitrogen; AP, available phosphorus; TK, total potassium; —, not determined.

**Table 5:** Major oxide composition of the three hydrochars determined by X-ray fluorescence (XRF) spectroscopy (wt.%).

Material	SiO_2_	K_2_O	CaO	P_2_O_5_	Fe_2_O_3_	Al_2_O_3_	MgO	SO_3_
Corncob hydrochar	7.97	2.41	4.53	0.97	0.56	0.85	0.73	0.91
Corn straw hydrochar	9.32	4.32	4.67	1.75	0.75	0.68	1.62	0.42
Rice husk hydrochar	17.72	5.38	2.35	2.57	0.67	0.72	0.89	0.68

Note: The XRF results are reported on an oxide-equivalent basis.

## Data Availability

The data presented in this study are available upon request from the corresponding author.

## Supplementary Materials

The following supporting information can be downloaded at: https://www.mdpi.com/article/10.3390/plants15203201/s1, Table S1: Comparison of the present study with representative studies on coal gangue, carbonaceous amendments, and arbuscular mycorrhizal fungi (AMF) in soil restoration; Table S2: Soil pH and electrical conductivity (EC) before and after ryegrass cultivation and the corresponding treatment-specific changes; Table S3: Repeated-measures ANOVA and simple-effects analyses of soil pH and EC before and after ryegrass cultivation; Table S4: Soil nutrient contents before and after ryegrass cultivation under different hydrochar treatments; Table S5: Within-treatment comparisons of soil nutrient contents before and after ryegrass cultivation; Table S6: Statistical analysis of ryegrass growth and root traits under different hydrochar treatments; Table S7: Ryegrass growth and root traits under different hydrochar treatments; Table S8: Blocked mixed-design repeated-measures ANOVA for soil nutrient contents in the rhizobox experiment; Table S9: Estimated marginal means of soil nutrient contents in the 0–10 cm layer under different treatments at the end of the four-month rhizobox experiment; Table S10: Tukey-adjusted pairwise comparisons of soil nutrient contents among treatments in the 0–10 cm layer at the end of the four-month rhizobox experiment; Table S11: Blocked mixed-design repeated-measures ANOVA for soil macroaggregate fractions in the rhizobox experiment; Table S12: Blocked simple-effects analyses of treatment effects on soil macroaggregate fractions within each sampling time × soil layer combination in the rhizobox experiment; Table S13: Estimated marginal means and Tukey groupings from blocked treatment analyses of soil macroaggregate fractions in the 0–10 cm layer at Month 4; Table S14: Tukey-adjusted pairwise comparisons from blocked treatment analyses of soil macroaggregate fractions in the 0–10 cm layer at Month 4; Table S15: Blocked mixed-design repeated-measures ANOVA for rhizobox soil water characteristics; Table S16: Blocked simple-effects analyses of treatment effects on rhizobox soil water characteristics within each sampling time × soil layer combination; Table S17: Estimated marginal means and Tukey groupings from blocked treatment analyses of rhizobox soil water characteristics in the 0–10 cm layer at Month 4; Table S18: Tukey-adjusted pairwise comparisons from blocked treatment analyses of rhizobox soil water characteristics in the 0–10 cm layer at Month 4; Table S19: Estimated marginal means and Tukey groupings from the blocked analysis of alfalfa maximum root length; Table S20: Tukey-adjusted pairwise comparisons from the blocked analysis of alfalfa maximum root length; Table S21: Total heavy metal concentrations in pot soil under different treatments (mg kg^−1^); Table S22: One-way ANOVA for total heavy metal concentrations in pot soil; Table S23: Factorial ANOVA of hydrochar type and application rate effects on total heavy metal concentrations in pot soil; Table S24: Follow-up simple-effects analysis for Ni in pot soil; Table S25: Blocked mixed-design repeated-measures ANOVA for total heavy metal concentrations in rhizobox soil; Table S26: Estimated marginal means of total heavy metal concentrations across soil layers from the blocked repeated-measures analysis of the rhizobox experiment; Table S27: Tukey-adjusted comparisons of amendment treatments with S for rhizobox soil heavy metal concentrations; Table S28: Follow-up analysis of significant soil-layer effects for Cr and Zn in the rhizobox experiment; Table S29: Heavy metal concentrations (mg kg^−1^) in shoots and roots of ryegrass under different treatments in the pot experiment; Table S30: Effects of treatment, plant organ, and their interaction on heavy metal concentrations in ryegrass; Table S31: Dunnett-adjusted comparisons of heavy metal concentrations in ryegrass shoots and roots between hydrochar treatments and the control in the pot experiment; Table S32: Organ-specific contrasts and effects of hydrochar type and application rate on heavy metal concentrations in ryegrass; Table S33: Heavy metal concentrations in alfalfa shoots and roots and their blocked treatment- and organ-dependent responses in the rhizobox experiment; Table S34: Planned comparisons from blocked analyses of treatment and organ effects on heavy metal concentrations in alfalfa in the rhizobox experiment; Table S35: Summary of major soil and plant responses across treatments in the pot and rhizobox experiments.
