# Supplementary material for: Hydrochar and Arbuscular Mycorrhizal Fungi Improve Soil Properties and Plant Growth in Coal Gangue-Amended Sandy Soil
Source: Plants (Basel). 2026 Oct 19;15(20):3201. doi: 10.3390/plants15203201 (PMC13708921; doi:10.3390/plants15203201)
Supplement: Supplementary file 1 [file plants-15-03201-s001.zip › plants-4595168-supplementary.pdf]

Table S1. Comparison of the present study with representative studies on coal gangue, carbonaceous amendments, and arbuscular mycorrhizal fungi (AMF) in soil restoration.

Study	Restoration context	Amendment strategy	Plant	Main responses evaluated	Main findings	Distinction from the present study
**Lu et al. [29]**	Arid mining-area substrate	Coal gangue + water-retaining agent + bentonite	*Kochia prostrata*	Substrate nutrients; germination; survival; plant growth	Amendment proportions affected substrate nutrient status and plant establishment and growth	Coal gangue-based substrate reconstruction; no hydrochar or AMF
**Ding et al. [11]**	Aeolian sandy soil	Coal gangue powder at 10 and 20%	*Medicago sativa*	Soil physical and hydraulic properties; emergence; photosynthesis; plant growth	Coal gangue improved soil water retention and supported alfalfa growth, with rate-dependent responses	Coal gangue amendment of sandy soil with alfalfa; no hydrochar or AMF
**Abel et al. [20]**	Sandy soils	Biochar and hydrochar at different rates	None	Bulk density; pore volume; water retention; available water capacity; wettability	Char amendments altered soil physical properties and generally increased available water capacity, with material- and soil-dependent responses	Hydrochar/biochar effects on sandy-soil physical properties; no coal gangue, plant response, or AMF
**Mau et al. [19]**	Sandy soil	Poultry-litter hydrochar produced at different temperatures and applied at different rates	Lettuce	Soil physical properties; water retention; fertility; nitrate leaching; plant growth	Hydrochar modified soil physical and water-related properties, reduced nitrate leaching, and produced rate-dependent plant responses	Hydrochar production-condition and rate responses in sandy soil; no coal gangue or AMF
**Xu et al. [12]**	Sandy soil	Food-waste-derived hydrochars at different rates	Grass	Pore structure; water retention; hydraulic conductivity;	Hydrochar increased water retention but produced contrasting responses in other hydraulic processes	Hydrochar hydraulic responses in sandy soil; no coal gangue or AMF

				sorptivity; infiltration; plant growth		
**George et al. [30]**	Soil–plant system	Spent brewer's yeast-derived hydrochar	*Medicago sativa*	Plant growth; AM colonization; nodulation; soil aggregation	Hydrochar increased soil aggregation but reduced plant growth and AM colonization under the tested conditions	Hydrochar–alfalfa–AM association; no coal gangue-amended sandy substrate or targeted AMF inoculation
**Ohsowski et al. [31]**	Nutrient-poor post-mine sandpit	Biochar + compost + AMF inoculum	Eight grassland species	Plant establishment and growth; plant cover; AMF colonization	Amendment responses depended on treatment combination, and AMF-containing treatments did not consistently improve plant performance	Carbonaceous amendment + AMF in post-mine sand; no hydrochar or coal gangue-amended substrate
**Zhang et al. [33]**	Sandy soil	Biochar, AMF, and biochar + AMF	*Nitraria sibirica*	Plant growth and nutrients; soil nutrients; water-stable aggregates; AMF colonization; microbial communities	Biochar and AMF produced treatment-dependent responses, while their combination increased larger water-stable aggregates and altered plant and microbial responses	Biochar + AMF in sandy soil; no coal gangue or hydrochar feedstock/rate screening
**Dong et al. [32]**	Coal-mining reclaimed soil	Biochar, AMF, and biochar + AMF	Maize	Soil structure and water retention; nutrients; enzymes; microbial communities; soil multifunctionality; plant production	Combined biochar and AMF improved soil structure, nutrient supply, microbial properties, soil multifunctionality, and maize production	Biochar + AMF in coal-mining reclaimed soil; no hydrochar feedstock/rate screening or coal gangue-amended sandy substrate

**Present study**	**Coal gangue-amended sandy soil**	**CCH, CSH, and RHH at 2–8%; selected hydrochars at 8%; RHH with or without AMF**	*Lolium perenne*; *Medicago sativa*	**Soil physicochemical and water-related properties; aggregation; plant and root traits; total metals; AMF colonization**	**Responses varied with hydrochar type and rate, with RHH showing the strongest overall performance; subsequent AMF inoculation was associated with additional improvements in several soil and plant responses**	**Hydrochar feedstock/rate screening in coal gangue-amended sandy soil, followed by soil-profile evaluation of selected hydrochars and assessment of the additional response to AMF inoculation after RHH amendment**

Note: AM, arbuscular mycorrhizal; AMF, arbuscular mycorrhizal fungi; CCH, corncob hydrochar; CSH, corn straw hydrochar; RHH, rice husk hydrochar. Reference numbers correspond to those in the main manuscript.

Table S2. Soil pH and electrical conductivity (EC) before and after ryegrass cultivation and the corresponding treatment-specific changes.

Treatment	pH before	pH after	ΔpH	EC before (μS cm⁻¹)	EC after (μS cm⁻¹)	ΔEC (μS cm⁻¹)
CK	7.62 ± 0.04	7.36 ± 0.05	0.260 ± 0.040 b	612.6 ± 6.8	370.6 ± 7.1	242.0 ± 12.8 def
CCH2	7.67 ± 0.06	7.29 ± 0.04	0.387 ± 0.065 ab	512.8 ± 11.8	248.6 ± 8.6	264.2 ± 3.3 e
CCH4	7.69 ± 0.05	7.33 ± 0.03	0.360 ± 0.062 ab	575.6 ± 9.9	278.5 ± 10.8	297.2 ± 1.8 d
CCH6	7.72 ± 0.02	7.34 ± 0.02	0.380 ± 0.010 ab	604.7 ± 14.0	284.0 ± 14.2	320.7 ± 3.3 c
CCH8	7.77 ± 0.05	7.40 ± 0.06	0.370 ± 0.105 ab	634.6 ± 10.2	312.8 ± 11.8	321.7 ± 1.7 c
CSH2	7.61 ± 0.07	7.22 ± 0.12	0.397 ± 0.049 ab	593.7 ± 6.5	273.3 ± 7.3	320.4 ± 1.8 c
CSH4	7.67 ± 0.03	7.23 ± 0.03	0.443 ± 0.025 a	648.5 ± 9.5	286.7 ± 10.6	361.8 ± 3.6 b
CSH6	7.68 ± 0.02	7.27 ± 0.05	0.407 ± 0.046 a	676.9 ± 11.4	296.7 ± 8.5	380.2 ± 5.3 b
CSH8	7.82 ± 0.03	7.37 ± 0.02	0.453 ± 0.012 a	687.3 ± 11.8	304.2 ± 14.2	383.1 ± 11.8 bc
RHH2	7.77 ± 0.02	7.32 ± 0.03	0.443 ± 0.021 a	527.0 ± 10.7	297.5 ± 9.3	229.5 ± 1.4 f
RHH4	7.89 ± 0.03	7.44 ± 0.04	0.447 ± 0.031 a	646.3 ± 9.3	313.2 ± 12.1	333.1 ± 4.6 c
RHH6	7.91 ± 0.02	7.45 ± 0.01	0.457 ± 0.015 a	733.9 ± 10.9	341.3 ± 5.7	392.6 ± 13.6 bc
RHH8	7.98 ± 0.01	7.51 ± 0.04	0.470 ± 0.035 a	847.6 ± 11.2	351.7 ± 4.8	495.9 ± 10.8 a

Note: Values are presented as mean ± standard deviation (SD) (n = 3 independent pots per treatment). ΔpH and ΔEC were calculated for each pot as the pre-planting value minus the corresponding post-planting value. Treatment effects on ΔpH were evaluated using one-way analysis of variance (ANOVA) followed by Tukey's honestly significant difference (HSD) test. Because the homogeneity-of-variance assumption was not satisfied for ΔEC (Levene's test, P = 0.013), ΔEC was analyzed using Welch's ANOVA followed by Games–Howell multiple comparisons. Within each change-score column, treatments sharing at least one lowercase letter are not significantly different, whereas treatments without a common letter differ significantly at P < 0.05. CK, control without hydrochar amendment; CCH, corncob hydrochar; CSH, corn straw hydrochar; RHH, rice husk hydrochar; numbers following the abbreviations indicate hydrochar application rates (%).

Table S3. Repeated-measures ANOVA and simple-effects analyses of soil pH and EC before and after ryegrass cultivation.

**Indicator**	**Analysis/effect**	**df**	**F**	**P**	**partial η^2**	**90% CI of partial η^2**
pH	Treatment	12, 26	20.32	<0.001	0.904	0.82–0.94
pH	Time	1, 26	2870.00	<0.001	0.991	0.986–0.994
pH	Treatment × Time	12, 26	4.38	<0.001	0.669	0.40–0.79
EC	Treatment	12, 26	103.12	<0.001	0.979	0.96–0.99
EC	Time	1, 26	80987.84	<0.001	0.9997	0.9995–0.9999
EC	Treatment × Time	12, 26	284.31	<0.001	0.9924	0.986–0.996
pH	Treatment at pre-planting	12, 26	26.78	<0.001	0.925	0.87–0.95
pH	Treatment at post-planting	12, 26	9.20	<0.001	0.809	0.68–0.88
EC	Treatment at pre-planting	12, 26	210.59	<0.001	0.990	0.98–0.995
EC	Treatment at post-planting	12, 26	33.96	<0.001	0.940	0.89–0.97

Note: A two-way mixed-design repeated-measures ANOVA was performed with treatment as the between-subject factor and time (before vs. after planting) as the within-subject factor. Each pot was treated as an independent experimental unit (n = 3 pots per treatment). Partial η^2 and its 90% confidence interval (CI) are reported as measures of effect size. Because time contained only two levels, no sphericity correction was required. Simple effects of treatment were examined separately at each time point to characterize significant treatment × time interactions.

Table S4. Soil nutrient contents before and after ryegrass cultivation under different hydrochar treatments.

Treatment	AP before	AP after	AK before	AK after	AH-N before	AH-N after	SOM before	SOM after
CK	6.39±0.85	1.88±0.85 h	8.33±0.73	2.70±0.79 h	32.50±0.70	22.03±0.75 g	13.84±0.65	14.81±0.70 f
CCH2	15.41±0.80	12.87±0.83 g	19.55±0.75	15.93±0.83 g	42.00±0.80	22.75±0.85 g	19.25±0.75	20.56±0.75 d
CCH4	18.72±0.80	13.92±0.83 efg	25.02±0.80	16.76±0.85 fg	42.00±0.70	28.00±0.80 f	18.56±0.75	19.69±0.75 de
CCH6	23.85±0.85	15.42±0.83 cdef	31.65±0.80	18.77±0.83 cdef	45.50±0.80	38.50±0.75 b	25.48±0.73	26.51±0.80 c
CCH8	29.40±0	21.36±0	38.01±0	26.62±0	52.00±0	48.27±0	27.20±0	28.34±0

	.85	.85 a	.80	.83 a	.80	.78 a	.75	.80 bc
CSH2	14.88±0.83	13.25±0.85 fg	18.13±0.78	16.10±0.85 g	35.00±0.80	33.25±0.80 de	16.52±0.73	17.52±0.73 e
CSH4	16.53±0.83	14.15±0.85 efg	20.21±0.80	16.43±0.83 fg	41.00±0.80	35.25±0.80 cd	25.14±0.75	26.51±0.80 c
CSH6	19.75±0.85	14.98±0.83 defg	24.90±0.80	17.60±0.85 defg	44.98±0.83	37.27±0.83 bc	28.25±0.75	28.85±0.80 b
CSH8	25.15±0.85	16.82±0.83 cd	32.34±0.83	19.95±0.85 cd	48.50±0.80	39.25±0.80 b	31.00±0.80	32.84±0.80 a
RHH2	16.27±0.83	14.55±0.85 defg	19.51±0.80	17.27±0.83 efg	35.00±0.80	22.75±0.85 g	19.95±0.75	20.52±0.73 d
RHH4	21.55±0.85	15.83±0.88 cde	26.35±0.85	19.27±0.83 cde	35.00±0.80	31.50±0.80 e	20.65±0.75	21.50±0.80 d
RHH6	24.90±0.85	17.52±0.83 bc	31.08±0.83	21.12±0.83 c	38.50±0.80	31.52±0.83 e	29.63±0.78	30.43±0.78 b
RHH8	30.11±0.85	19.65±0.85 ab	37.93±0.83	24.12±0.88 b	42.00±0.80	38.50±0.80 b	31.32±0.78	32.85±0.80 a

Note: Values are mean ± SD (n = 3 independent pots per treatment). AP, available phosphorus; AK, available potassium; AH-N, alkali-hydrolyzable nitrogen; SOM, soil organic matter. Different lowercase letters within each post-planting column indicate significant differences among treatments according to Tukey's HSD test ($P < 0.05$); values sharing at least one letter are not significantly different.

Table S5. Within-treatment comparisons of soil nutrient contents before and after ryegrass cultivation.

Variable	Treatment	Difference (post − pre)	t	Holm-adjusted P
AP	CK	−4.517	−4.962	0.2535
AP	CCH2	−2.540	−2.707	0.4547
AP	CCH4	−4.797	−5.111	0.2535
AP	CCH6	−8.430	−8.717	0.1549
AP	CCH8	−8.047	−8.198	0.1549
AP	CSH2	−1.633	−1.689	0.4547
AP	CSH4	−2.377	−2.458	0.4547
AP	CSH6	−4.777	−4.939	0.2535
AP	CSH8	−8.323	−8.607	0.1549
AP	RHH2	−1.720	−1.779	0.4547
AP	RHH4	−5.727	−5.750	0.2315
AP	RHH6	−7.373	−7.624	0.1549
AP	RHH8	−10.460	−10.657	0.1130
AK	CK	−5.630	−6.436	0.1165
AK	CCH2	−3.620	−3.981	0.2258
AK	CCH4	−8.260	−8.670	0.1043
AK	CCH6	−12.873	−13.721	0.0652

AK	CCH8	−11.387	−12.136	0.0672
AK	CSH2	−2.030	−2.164	0.2806
AK	CSH4	−3.780	−4.029	0.2258
AK	CSH6	−7.303	−7.666	0.1161
AK	CSH8	−12.390	−12.810	0.0664
AK	RHH2	−2.233	−2.380	0.2806
AK	RHH4	−7.073	−7.314	0.1161
AK	RHH6	−9.963	−10.458	0.0812
AK	RHH8	−13.810	−14.070	0.0652
AH-N	CK	−10.467	−12.500	0.0659
AH-N	CCH2	−19.250	−20.207	0.0317
AH-N	CCH4	−14.000	−16.166	0.0457
AH-N	CCH6	−7.000	−7.822	0.1192
AH-N	CCH8	−3.733	−4.105	0.2181
AH-N	CSH2	−1.750	−1.894	0.2181
AH-N	CSH4	−5.750	−6.225	0.1243
AH-N	CSH6	−7.717	−8.099	0.1192
AH-N	CSH8	−9.250	−10.013	0.0884
AH-N	RHH2	−12.250	−12.859	0.0659
AH-N	RHH4	−3.500	−3.789	0.2181
AH-N	RHH6	−6.983	−7.443	0.1192
AH-N	RHH8	−3.500	−3.789	0.2181
SOM	CK	+0.967	29.000	0.0047
SOM	CCH2	+1.303	391.000	<0.001
SOM	CCH4	+1.137	85.250	0.0011
SOM	CCH6	+1.023	23.615	0.0047
SOM	CCH8	+1.143	38.591	0.0040
SOM	CSH2	+1.000	>999†	<0.001
SOM	CSH4	+1.363	42.875	0.0038
SOM	CSH6	+0.603	20.762	0.0047
SOM	CSH8	+1.840	184.000	<0.001
SOM	RHH2	+0.570	37.315	0.0040
SOM	RHH4	+0.857	28.915	0.0047
SOM	RHH6	+0.807	121.000	<0.001
SOM	RHH8	+1.523	104.843	<0.001

Note: Differences were calculated as post-planting minus pre-planting values measured in the same independent pots (n = 3 per treatment). AP, available phosphorus; AK, available potassium; AH-N, alkali-hydrolyzable nitrogen; SOM, soil organic matter. Within each treatment, pre- and post-planting values were compared using paired t-tests. P values were adjusted using the Holm procedure separately for each nutrient variable across the 13 treatment-wise comparisons to control the family-wise error rate. Holm-adjusted $P < 0.05$ was considered statistically significant. †For SOM in CSH2, all three paired differences were identical (+1.00 g kg^{-1}), resulting in zero variance of the paired differences; the t statistic therefore tends to infinity and is reported as >999.

Table S6. Statistical analysis of ryegrass growth and root traits under different hydrochar treatments.

Trait	Scale analyzed	Shapiro-Wilk p	Levene P	F(12,26)	P	η^2	ω^2
Plant height	Raw	0.529	0.974	120.04	<0.001	0.982	0.973
Root length	Raw	0.084	0.938	103.80	<0.001	0.980	0.969
Fresh weight	Log	0.125	0.987	84.04	<0.001	0.975	0.962
Dry weight	Raw	0.297	0.994	67.27	<0.001	0.969	0.953
Root surface area	Log	0.674	0.930	2442.62	<0.001	0.999	0.999
Root volume	Raw	0.635	0.963	135.21	<0.001	0.984	0.976
Average root diameter	Raw	0.567	0.898	26.32	<0.001	0.924	0.886

Note: Each pot was treated as an independent experimental unit (n = 3 pots per treatment). Treatment effects were evaluated using one-way ANOVA followed by Tukey's HSD test. Model assumptions were assessed using the Shapiro–Wilk test on ANOVA residuals and Levene's test for homogeneity of variances. Fresh weight and root surface area were log-transformed before inferential analysis because residual normality was not satisfied on the original scale; the transformed data satisfied the evaluated assumptions. η^2 and ω^2 represent treatment effect sizes. All other variables were analyzed on the original measurement scale.

Table S7. Ryegrass growth and root traits under different hydrochar treatments.

Treatment	Plant height (cm)	Root length (cm)	Fresh weight (g)	Dry weight (g)	Root surface area (cm^2)	Root volume (cm^3)	Average root diameter (mm)
CK	14.53 ± 0.50 f	7.50 ± 0.60 f	5.33 ± 0.45 i	3.60 ± 0.56 h	72.37 ± 1.67 i	11.73 ± 0.96 j	0.97 ± 0.21 g
CCH2	18.57 ± 0.40 e	20.23 ± 0.87 e	8.37 ± 0.55 h	7.23 ± 0.59 g	132.37 ± 2.30 g	27.20 ± 1.30 gh	1.60 ± 0.10 def
CCH4	27.43 ± 1.30 bc	25.40 ± 1.35 bcd	9.50 ± 0.40 fgh	8.63 ± 0.67 efg	148.80 ± 2.17 f	30.93 ± 1.43 fg	1.67 ± 0.15 def
CCH6	28.27 ± 0.93 bc	26.33 ± 1.43 abcd	10.37 ± 0.55 efg	9.20 ± 0.62 def	159.43 ± 1.98 e	32.97 ± 2.78 ef	1.73 ± 0.15 de

CCH8	28.63 ± 0.65 abc	27.13 ± 0.71 abc	11.20 ± 0.70 def	9.50 ± 0.36 de	165.07 ± 2.68 e	39.63 ± 1.30 cd	1.90 ± 0.10 cde
CSH2	24.27 ± 0.55 d	24.43 ± 0.93 cd	8.53 ± 0.60 h	6.80 ± 0.66 g	92.40 ± 2.07 h	21.73 ± 1.57 i	1.20 ± 0.10 fg
CSH4	27.90 ± 0.56 bc	25.37 ± 0.81 bcd	9.17 ± 0.50 gh	7.33 ± 0.81 fg	158.50 ± 1.81 e	22.77 ± 1.80 hi	1.40 ± 0.10 efg
CSH6	28.43 ± 0.50 bc	26.47 ± 1.27 abcd	11.50 ± 0.36 cde	10.20 ± 0.46 cde	213.63 ± 1.66 c	41.53 ± 0.75 abc	2.00 ± 0.10 bcd
CSH8	28.93 ± 0.60 abc	27.63 ± 1.21 ab	12.30 ± 0.66 cd	10.63 ± 0.76 cd	222.53 ± 2.35 c	45.07 ± 1.06 ab	2.40 ± 0.10 abc
RHH2	26.83 ± 0.95 c	24.13 ± 0.15 d	12.10 ± 0.80 cde	10.23 ± 0.59 cde	184.57 ± 2.30 d	29.43 ± 1.36 fg	1.80 ± 0.20 de
RHH4	28.10 ± 0.75 bc	25.13 ± 0.59 bcd	13.37 ± 0.74 bc	11.83 ± 1.00 bc	251.63 ± 1.86 b	35.77 ± 1.70 de	1.93 ± 0.15 bcd
RHH6	29.27 ± 0.75 ab	26.83 ± 0.31 abcd	15.23 ± 0.45 ab	13.57 ± 0.47 ab	260.93 ± 2.06 b	40.93 ± 1.50 bc	2.43 ± 0.32 ab
RHH8	**30.67 ± 0.59 a**	**28.57 ± 0.76 a**	**17.23 ± 0.70 a**	**14.97 ± 0.31 a**	**291.23 ± 2.06 a**	**45.80 ± 1.05 a**	**2.90 ± 0.30 a**

Note: Values are presented as mean ± SD (n = 3 independent pots per treatment). Each pot, rather than individual plants within a pot, was treated as the independent experimental unit. Different lowercase letters within the same column indicate significant differences among treatments according to Tukey's HSD test ($P < 0.05$); values sharing at least one letter are not significantly different. Fresh weight and root surface area were log-transformed before one-way ANOVA and Tukey's HSD testing to satisfy model assumptions, whereas descriptive values are presented on the original measurement scale. CCH, corncob hydrochar; CSH, corn straw hydrochar; RHH, rice husk hydrochar; numbers following the abbreviations indicate hydrochar application rates (%).

Table S8. Blocked mixed-design repeated-measures ANOVA for soil nutrient contents in the rhizobox experiment.

Source	df	AH-N, F (partial η^2)	AP, F (partial η^2)	AK, F (partial η^2)	SOM, F (partial η^2)
Treatment	4, 8	2715.01*** (0.9993)	8305.89*** (0.9998)	5215.18*** (0.9996)	2485.53*** (0.9992)
Layer	2, 20	4486.38*** (0.9978)	4829.98*** (0.9979)	1888.23*** (0.9947)	1997.39*** (0.9950)
Time	1, 10	348.13*** (0.9721)	618.79*** (0.9841)	474.55*** (0.9794)	374.46*** (0.9740)
Treatment × Layer	8, 20	8.50*** (0.7727)	318.34*** (0.9922)	520.84*** (0.9952)	223.35*** (0.9889)

Treatment × Time	4, 10	12.18*** (0.8297)	30.81*** (0.9249)	112.58*** (0.9783)	21.15*** (0.8943)
Layer × Time	2, 20	679.48*** (0.9855)	249.76*** (0.9615)	731.80*** (0.9865)	276.59*** (0.9651)
Treatment × Layer × Time	8, 20	45.94*** (0.9484)	24.28*** (0.9066)	217.78*** (0.9887)	20.64*** (0.8920)

Note: The treatment-specific compartment was the experimental unit, with each treatment represented by three compartments, one in each rhizobox (n = 3). Rhizobox identity was included as a blocking factor. Treatment was the fixed between-compartment factor, whereas soil layer and sampling time were repeated within-compartment factors. The treatment effect was evaluated using the between-compartment error after accounting for rhizobox blocking, whereas soil layer, sampling time, and their interactions were evaluated using the corresponding repeated-measures error strata. Soil layers were 0 – 10, 10 – 20, and 20 – 30 cm, and sampling times were Month 1 and Month 4. Values are F statistics with partial η^2 in parentheses. AH-N, alkali-hydrolyzable nitrogen; AP, available phosphorus; AK, available potassium; SOM, soil organic matter. *** $P < 0.001$.

Table S9. Estimated marginal means of soil nutrient contents in the 0 – 10 cm layer under different treatments at the end of the four-month rhizobox experiment.

Indicator	Treatment	EMM	95% CI	Tukey group
AH-N	CK	34.740	34.469–35.013	e
	S	42.690	42.417–42.963	d
	S-CSH	51.587	51.313–51.860	c
	S-RHH	54.733	54.460–55.007	b
	S-RHH-AMF	72.473	72.200–72.747	a
AP	CK	36.480	33.975–38.985	d
	S	56.507	54.002–59.012	c
	S-CSH	74.370	71.865–76.875	b
	S-RHH	77.697	75.192–80.202	b
	S-RHH-AMF	90.530	88.025–93.035	a
AK	CK	3.063	2.321–3.805	e

	S	8.690	7.948–9.432	d
	S-CSH	13.690	12.948–14.432	c
	S-RHH	20.183	19.441–20.925	b
	S-RHH-AMF	39.453	38.711–40.195	a
SOM	CK	16.520	14.706–18.334	d
	S	29.240	27.426–31.054	c
	S-CSH	34.667	32.853–36.481	b
	S-RHH	38.100	36.286–39.914	b
	S-RHH-AMF	53.473	51.659–55.287	a

Note: Values are estimated marginal means (EMMs) from blocked treatment analyses for the 0 – 10 cm soil layer at Month 4, with treatment as the fixed factor and rhizobox identity included as the blocking factor. Each treatment was represented by three treatment compartments, one in each rhizobox (n = 3). Different lowercase letters within each nutrient indicate significant differences among treatments based on Tukey-adjusted comparisons (P < 0.05). CK, unamended sandy soil control; S, coal gangue:sandy soil = 4:6 (w/w); CSH, corn straw hydrochar; RHH, rice husk hydrochar; S-CSH, S amended with 8% CSH (w/w); S-RHH, S amended with 8% RHH (w/w); S-RHH-AMF, S-RHH inoculated with arbuscular mycorrhizal fungi (AMF); AH-N, alkali-hydrolyzable nitrogen; AP, available phosphorus; AK, available potassium; SOM, soil organic matter.

Table S10. Tukey-adjusted pairwise comparisons of soil nutrient contents among treatments in the 0 – 10 cm layer at the end of the four-month rhizobox experiment.

**Indicator**	**Comparison**	**Mean difference**	**95%CI**	**Tukey-adjusted P**
AH-N	S-RHH-AMF vs S-RHH	17.740	17.161–18.319	<0.001
AH-N	S-RHH-AMF vs S-CSH	20.887	20.307–21.466	<0.001
AH-N	S-RHH-AMF vs S	29.783	29.204–30.363	<0.001
AH-N	S-RHH-AMF vs CK	37.733	37.154–38.313	<0.001
AH-N	S-RHH vs S-CSH	3.147	2.567–3.726	<0.001
AH-N	S-RHH vs S	12.043	11.464–12.623	<0.001

AH-N	S-RHH vs CK	19.993	19.414–20.573	<0.001
AH-N	S-CSH vs S	8.897	8.317–9.476	<0.001
AH-N	S-CSH vs CK	16.847	16.267–17.426	<0.001
AH-N	S vs CK	7.950	7.371–8.529	<0.001
AP	S-RHH-AMF vs S-RHH	12.833	7.526–18.141	0.0002
AP	S-RHH-AMF vs S-CSH	16.160	10.853–21.467	<0.001
AP	S-RHH-AMF vs S	34.023	28.716–39.331	<0.001
AP	S-RHH-AMF vs CK	54.050	48.743–59.357	<0.001
AP	S-RHH vs S-CSH	3.327	−1.981–8.634	0.2819
AP	S-RHH vs S	21.190	15.883–26.497	<0.001
AP	S-RHH vs CK	41.217	35.909–46.524	<0.001
AP	S-CSH vs S	17.863	12.556–23.171	<0.001
AP	S-CSH vs CK	37.890	32.583–43.197	<0.001
AP	S vs CK	20.027	14.719–25.334	<0.001
AK	S-RHH-AMF vs S-RHH	19.270	17.698–20.842	<0.001
AK	S-RHH-AMF vs S-CSH	25.763	24.191–27.335	<0.001
AK	S-RHH-AMF vs S	30.763	29.191–32.335	<0.001
AK	S-RHH-AMF vs CK	36.390	34.818–37.962	<0.001
AK	S-RHH vs S-CSH	6.493	4.921–8.065	<0.001
AK	S-RHH vs S	11.493	9.921–13.065	<0.001
AK	S-RHH vs CK	17.120	15.548–18.692	<0.001
AK	S-CSH vs S	5.000	3.428–6.572	<0.001
AK	S-CSH vs CK	10.627	9.055–12.199	<0.001
AK	S vs CK	5.627	4.055–7.199	<0.001
SOM	S-RHH-AMF vs S-RHH	15.373	11.530–19.217	<0.001
SOM	S-RHH-AMF vs S-CSH	18.807	14.963–22.650	<0.001

SOM	S-RHH-AMF vs S	24.233	20.390–28.077	<0.001
SOM	S-RHH-AMF vs CK	36.953	33.110–40.797	<0.001
SOM	S-RHH vs S-CSH	3.433	−0.410–7.277	0.0827
SOM	S-RHH vs S	8.860	5.017–12.703	0.0003
SOM	S-RHH vs CK	21.580	17.737–25.423	<0.001
SOM	S-CSH vs S	5.427	1.583–9.270	0.0079
SOM	S-CSH vs CK	18.147	14.303–21.990	<0.001
SOM	S vs CK	12.720	8.877–16.563	<0.001

Note: Pairwise treatment contrasts were obtained from the blocked treatment analyses for the 0 – 10 cm soil layer at Month 4, with treatment as the fixed factor and rhizobox identity included as the blocking factor. Mean differences are expressed as the first treatment minus the second treatment. Confidence intervals and P values were adjusted for multiple comparisons among the five treatments using Tukey's procedure. Each treatment was represented by three treatment compartments, one in each rhizobox (n = 3). P values below 0.001 are reported as P < 0.001. AH-N, alkali-hydrolyzable nitrogen; AP, available phosphorus; AK, available potassium; SOM, soil organic matter.

Table S11. Blocked mixed-design repeated-measures ANOVA for soil macroaggregate fractions in the rhizobox experiment.

Aggregate indicator	Source	df	F	P	partial η²
Dry-sieved ≥0.5 mm fraction (%)	Treatment	4, 8	18028.423	<0.001	0.9999
	Soil layer	2, 20	89.879	<0.001	0.8999
	Time	1, 10	6084.166	<0.001	0.9984
	Treatment × Soil layer	8, 20	7.163	<0.001	0.7413
	Treatment × Time	4, 10	21.463	<0.001	0.8957
	Soil layer × Time	2, 20	23.939	<0.001	0.7054
	Treatment × Soil layer × Time	8, 20	9.603	<0.001	0.7934
Water-stable ≥0.5 mm fraction (%)	Treatment	4, 8	8394.081	<0.001	0.9998
	Soil layer	2, 20	47.942	<0.001	0.8274
	Time	1, 10	59312.218	<0.001	0.9998
	Treatment × Soil layer	8, 20	21.686	<0.001	0.8966
	Treatment × Time	4, 10	1028.686	<0.001	0.9976
	Soil layer × Time	2, 20	10.419	<0.001	0.5103
	Treatment × Soil layer × Time	8, 20	1.828	0.131	0.4224

Note: The treatment-specific compartment was the experimental unit, with each treatment represented

by three compartments, one in each rhizobox (n = 3). Rhizobox identity was included as a blocking factor. Treatment was the fixed between-compartment factor, whereas soil layer and sampling time were repeated within-compartment factors. The treatment effect was tested against the rhizobox × treatment error term, whereas soil layer, sampling time, and their interactions were tested against the corresponding compartment-by-within-factor repeated-measures error terms. Rhizobox-related terms were not of primary inferential interest and are therefore not shown. Soil layers were 0 – 10, 10 – 20, and 20 – 30 cm, and sampling times were Month 1 and Month 4. Partial η^2 is reported as the effect-size measure.

Table S12. Blocked simple-effects analyses of treatment effects on soil macroaggregate fractions within each sampling time × soil layer combination in the rhizobox experiment.

Aggregate indicator	Sampling time	Soil layer (cm)	F(4,8)	P	Partial η^2	ω^2
Dry-sieved ≥0.5 mm fraction (%)	Month 1	0–10	3145.54	<0.001	0.999	0.999
	Month 1	10–20	674.81	<0.001	0.997	0.995
	Month 1	20–30	1943.14	<0.001	0.999	0.998
	Month 4	0–10	447.39	<0.001	0.996	0.993
	Month 4	10–20	1432.71	<0.001	0.999	0.997
	Month 4	20–30	955.83	<0.001	0.998	0.996
Water-stable ≥0.5 mm fraction (%)	Month 1	0–10	1737.49	<0.001	0.999	0.998
	Month 1	10–20	1605.10	<0.001	0.999	0.998
	Month 1	20–30	4643.18	<0.001	1.000	0.999
	Month 4	0–10	2028.96	<0.001	0.999	0.998
	Month 4	10–20	843.20	<0.001	0.998	0.996
	Month 4	20–30	1433.03	<0.001	0.999	0.997

Note: Treatment simple effects were tested separately within each sampling time × soil layer combination, with treatment as the fixed factor and rhizobox identity included as the blocking factor. The treatment-specific compartment was the experimental unit; each treatment was represented by three compartments, one in each rhizobox (n = 3). Treatment effects were tested with F(4,8). Partial η^2 and ω^2 are reported as effect-size measures.

Table S13. Estimated marginal means and Tukey groupings from blocked treatment analyses of soil macroaggregate fractions in the 0 – 10 cm layer at Month 4.

Aggregate indicator	Treatment	n	EMM (%)	95% CI	Tukey group
Dry-sieved ≥0.5 mm fraction	CK	3	17.922	16.910–18.934	d

	S	3	27.503	26.491–28.515	c
	S-CSH	3	32.973	31.961–33.985	b
	S-RHH	3	33.857	32.845–34.869	b
	S-RHH-AMF	3	43.223	42.211–44.235	a
Water-stable ⩾0.5 mm fraction	CK	3	19.649	18.974–20.324	e
	S	3	52.443	51.768–53.118	a
	S-CSH	3	44.537	43.862–45.212	d
	S-RHH	3	46.470	45.795–47.145	c
	S-RHH-AMF	3	49.900	49.225–50.575	b

Note: Values are estimated marginal means (EMMs) from blocked treatment analyses for the 0 – 10 cm soil layer at Month 4, with treatment as the fixed factor and rhizobox identity included as the blocking factor. Each treatment was represented by three treatment compartments, one in each rhizobox (n = 3). Different lowercase letters within each aggregate indicator indicate significant differences among treatments based on Tukey-adjusted comparisons (P < 0.05).

Table S14. Tukey-adjusted pairwise comparisons from blocked treatment analyses of soil macroaggregate fractions in the 0 – 10 cm layer at Month 4.

**Aggregate indicator**	**Comparison**	**Mean difference (percentage points)**	**95%CI**	**Adjusted P**
Dry-sieved ≥0.5 mm fraction	S-RHH-AMF vs. S-RHH	9.366	7.222–11.510	<0.001
	S-RHH-AMF vs. S-CSH	10.250	8.106–12.394	<0.001
	S-RHH-AMF vs. S	15.720	13.576–17.864	<0.001
	S-RHH-AMF vs. CK	25.301	23.157–27.445	<0.001
	S-RHH vs. S-CSH	0.884	−1.260–3.028	0.6309
	S-RHH vs. S	6.354	4.210–8.498	0.0001
	S-RHH vs. CK	15.935	13.791–18.079	<0.001
	S-CSH vs. S	5.470	3.326–7.614	0.0002
	S-CSH vs. CK	15.051	12.907–17.195	<0.001

	S vs. CK	9.581	7.437–11.725	<0.001
Water-stable ≥0.5 mm fraction	S vs. S-RHH-AMF	2.543	1.113–3.974	0.0018
	S vs. S-RHH	5.973	4.543–7.404	<0.001
	S vs. S-CSH	7.907	6.476–9.337	<0.001
	S vs. CK	32.794	31.364–34.225	<0.001
	S-RHH-AMF vs. S-RHH	3.430	2.000–4.860	0.0002
	S-RHH-AMF vs. S-CSH	5.363	3.933–6.794	<0.001
	S-RHH-AMF vs. CK	30.251	28.821–31.682	<0.001
	S-RHH vs. S-CSH	1.933	0.503–3.364	0.0102
	S-RHH vs. CK	26.821	25.391–28.252	<0.001
	S-CSH vs. CK	24.888	23.457–26.318	<0.001

Note: Pairwise comparisons were obtained from the blocked treatment analyses for the 0–10 cm soil layer at Month 4, with treatment as the fixed factor and rhizobox identity included as the blocking factor. Mean differences are expressed in percentage points and correspond to the first treatment minus the second treatment. Confidence intervals and P values were adjusted for multiple comparisons among the five treatments using Tukey's procedure. Each treatment was represented by three treatment compartments, one in each rhizobox (n = 3). P values below 0.001 are reported as P < 0.001.

Table S15. Blocked mixed-design repeated-measures ANOVA for rhizobox soil water characteristics.

**Source**	**df**	**Sat.WC, F (partial η²)**	**FC, F (partial η²)**	**TP, F (partial η²)**	**CP, F (partial η²)**
Treatment	4, 8	105318.92*** (0.99998)	15944.64*** (0.99987)	54450.38*** (0.99996)	7266.29*** (0.99972)
Soil layer	2, 20	147.85*** (0.937)	103.03*** (0.912)	42.96*** (0.811)	14.87*** (0.598)
Time	1, 10	13770.30*** (0.999)	3058.42*** (0.997)	4635.71*** (0.998)	4100.10*** (0.998)
Treatment × Soil layer	8, 20	78.95*** (0.969)	116.84*** (0.979)	278.92*** (0.991)	127.38*** (0.981)
Treatment × Time	4, 10	1592.89*** (0.998)	233.12*** (0.989)	521.48*** (0.995)	599.53*** (0.996)
Soil layer × Time	2, 20	707.38*** (0.986)	853.20*** (0.988)	221.79*** (0.957)	214.61*** (0.956)

Treatment × Soil layer × Time	8, 20	138.08*** (0.982)	175.36*** (0.986)	116.16*** (0.979)	89.18*** (0.973)

Note: The treatment-specific compartment was the experimental unit, with each treatment represented by three compartments, one in each rhizobox (n = 3). Rhizobox identity was included as a blocking factor. Treatment was the fixed between-compartment factor, whereas soil layer and sampling time were repeated within-compartment factors. The treatment effect was evaluated using the between-compartment error after accounting for rhizobox blocking, whereas soil layer, sampling time, and their interactions were evaluated using the corresponding repeated-measures error strata. Soil layers were 0–10, 10–20, and 20–30 cm, and sampling times were Month 1 and Month 4. Values are F statistics with partial η^2 in parentheses. Sat.WC, saturated water content; FC, field capacity; TP, total porosity; CP, capillary porosity. *** P < 0.001.

Table S16. Blocked simple-effects analyses of treatment effects on rhizobox soil water characteristics within each sampling time × soil layer combination.

indicator	Sampling time	Soil layer	$F_{treatment}$(4,8)	$P_{treatment}$	$F_{rhizobox}$(2,8)	$P_{rhizobox}$	partial η^2	ω²
Sat.WC	Month 1	0–10 cm	15291.439	1.462×10^{-15}	111.79	1.424×10^{-6}	1.000	0.996
Sat.WC	Month 1	10–20 cm	12127.810	3.695×10^{-15}	89.75	3.314×10^{-6}	1.000	0.996
Sat.WC	Month 1	20–30 cm	14096.110	2.025×10^{-15}	69.26	8.888×10^{-6}	1.000	0.997
Sat.WC	Month 4	0–10 cm	22333.550	3.214×10^{-16}	110.95	1.466×10^{-6}	1.000	0.997
Sat.WC	Month 4	10–20 cm	62946.490	5.095×10^{-18}	365.49	1.373×10^{-8}	1.000	0.997
Sat.WC	Month 4	20–30 cm	10430.298	6.753×10^{-15}	57.57	1.781×10^{-5}	1.000	0.997
FC	Month 1	0–10 cm	1967.220	5.316×10^{-12}	120.35	1.071×10^{-6}	0.999	0.969
FC	Month 1	10–20 cm	4363.180	2.203×10^{-13}	209.29	1.237×10^{-7}	1.000	0.976
FC	Month 1	20–30 cm	3723.660	4.150×10^{-13}	200.91	1.452×10^{-7}	0.999	0.973
FC	Month 4	0–10	4017.040	3.066×10^{-13}	88.74	3.460×10^{-6}	1.000	0.988

		cm						
FC	Month 4	10–20 cm	1886.590	6.283×10⁻¹²	117.27	1.184×10⁻⁶	0.999	0.968
FC	Month 4	20–30 cm	3068.480	8.996×10⁻¹³	121.07	1.046×10⁻⁶	1.000	0.980
TP	Month 1	0–10 cm	2365.400	2.545×10⁻¹²	87.04	3.726×10⁻⁶	0.999	0.981
TP	Month 1	10–20 cm	2808.470	1.282×10⁻¹²	93.74	2.805×10⁻⁶	0.999	0.982
TP	Month 1	20–30 cm	4679.010	1.666×10⁻¹³	132.70	7.331×10⁻⁷	1.000	0.985
TP	Month 4	0–10 cm	14863.460	1.638×10⁻¹⁵	215.69	1.099×10⁻⁷	1.000	0.993
TP	Month 4	10–20 cm	6361.200	4.878×10⁻¹⁴	127.79	8.487×10⁻⁷	1.000	0.990
TP	Month 4	20–30 cm	6478.040	4.536×10⁻¹⁴	97.78	2.385×10⁻⁶	1.000	0.992
CP	Month 1	0–10 cm	219.930	3.274×10⁻⁸	90.58	3.200×10⁻⁶	0.991	0.819
CP	Month 1	10–20 cm	775.260	2.187×10⁻¹⁰	214.63	1.120×10⁻⁷	0.997	0.875
CP	Month 1	20–30 cm	508.880	1.171×10⁻⁹	102.47	1.992×10⁻⁶	0.996	0.903
CP	Month 4	0–10 cm	3803.160	3.814×10⁻¹³	153.72	4.137×10⁻⁷	0.999	0.979
CP	Month 4	10–20 cm	1569.010	1.312×10⁻¹¹	86.03	3.896×10⁻⁶	0.999	0.971
CP	Month 4	20–30 cm	459.820	1.753×10⁻⁹	67.80	9.633×10⁻⁶	0.996	0.925

Note: Treatment simple effects were tested separately within each sampling time × soil layer combination, with treatment as the fixed factor and rhizobox identity included as the blocking factor. The treatment-specific compartment was the experimental unit, and each treatment was represented by three compartments, one in each rhizobox ($n = 3$). Treatment effects were tested with $F(4,8)$, and rhizobox effects with $F(2,8)$. Partial η^2 and ω^2 refer to the treatment effect. Sat.WC, saturated water

content; FC, field capacity; TP, total porosity; CP, capillary porosity.

Table S17. Estimated marginal means and Tukey groupings from blocked treatment analyses of rhizobox soil water characteristics in the 0 – 10 cm layer at Month 4.

**Indicator**	**Treatment**	**EMM (%)**	**95% CI**	**Tukey group**
Sat.WC	CK	20.400	20.097–20.703	e
Sat.WC	S	35.507	35.204–35.809	d
Sat.WC	S-CSH	54.527	54.224–54.829	c
Sat.WC	S-RHH	60.497	60.194–60.799	b
Sat.WC	S-RHH-AMF	68.513	68.211–68.816	a
FC	CK	10.373	10.034–10.713	e
FC	S	14.797	14.457–15.136	d
FC	S-CSH	24.793	24.454–25.133	c
FC	S-RHH	26.580	26.241–26.919	b
FC	S-RHH-AMF	33.490	33.151–33.829	a
TP	CK	28.773	28.540–29.007	e
TP	S	32.627	32.393–32.860	d
TP	S-CSH	46.727	46.493–46.960	c
TP	S-RHH	49.623	49.390–49.857	b
TP	S-RHH-AMF	58.600	58.366–58.834	a
CP	CK	18.340	18.104–18.576	e
CP	S	20.503	20.267–20.740	d
CP	S-CSH	22.520	22.284–22.756	c
CP	S-RHH	25.690	25.454–25.926	b
CP	S-RHH-AMF	34.543	34.307–34.780	a

Note: Values are estimated marginal means (EMMs) from blocked treatment analyses for the 0 – 10 cm soil layer at Month 4, with treatment as the fixed factor and rhizobox identity included as the blocking factor. Each treatment was represented by three treatment compartments, one in each rhizobox (n = 3). Different lowercase letters within each soil water characteristic indicate significant differences among

treatments based on Tukey-adjusted comparisons (P < 0.05). Sat.WC, saturated water content; FC, field capacity; TP, total porosity; CP, capillary porosity.

Table S18. Tukey-adjusted pairwise comparisons from blocked treatment analyses of rhizobox soil water characteristics in the 0 – 10 cm layer at Month 4.

**Indicator**	**Comparison**	**Mean difference**	**95% CI**	**Tukey-adjusted P**
Sat.WC	S-RHH-AMF vs S-RHH	8.017	7.376–8.658	<0.001
Sat.WC	S-RHH-AMF vs S-CSH	13.987	13.346–14.628	<0.001
Sat.WC	S-RHH-AMF vs S	33.007	32.366–33.648	<0.001
Sat.WC	S-RHH-AMF vs CK	48.113	47.472–48.754	<0.001
Sat.WC	S-RHH vs S-CSH	5.970	5.329–6.611	<0.001
Sat.WC	S-RHH vs S	24.990	24.349–25.631	<0.001
Sat.WC	S-RHH vs CK	40.097	39.456–40.738	<0.001
Sat.WC	S-CSH vs S	19.020	18.379–19.661	<0.001
Sat.WC	S-CSH vs CK	34.127	33.486–34.768	<0.001
Sat.WC	S vs CK	15.107	14.466–15.748	<0.001
FC	S-RHH-AMF vs S-RHH	6.910	6.191–7.629	<0.001
FC	S-RHH-AMF vs S-CSH	8.697	7.978–9.416	<0.001
FC	S-RHH-AMF vs S	18.693	17.974–19.412	<0.001
FC	S-RHH-AMF vs CK	23.117	22.398–23.836	<0.001
FC	S-RHH vs S-CSH	1.787	1.068–2.506	0.0002
FC	S-RHH vs S	11.783	11.064–12.502	<0.001
FC	S-RHH vs CK	16.207	15.488–16.926	<0.001
FC	S-CSH vs S	9.997	9.278–10.716	<0.001
FC	S-CSH vs CK	14.420	13.701–15.139	<0.001
FC	S vs CK	4.423	3.704–5.142	<0.001
TP	S-RHH-AMF vs S-RHH	8.977	8.481–9.472	<0.001
TP	S-RHH-AMF vs S-CSH	11.873	11.378–12.369	<0.001
TP	S-RHH-AMF vs S	25.973	25.478–26.469	<0.001
TP	S-RHH-AMF vs CK	29.827	29.331–30.322	<0.001
TP	S-RHH vs S-CSH	2.897	2.401–3.392	<0.001
TP	S-RHH vs S	16.997	16.501–17.492	<0.001
TP	S-RHH vs CK	20.850	20.355–21.345	<0.001
TP	S-CSH vs S	14.100	13.605–14.595	<0.001
TP	S-CSH vs CK	17.953	17.458–18.449	<0.001
TP	S vs CK	3.853	3.358–4.349	<0.001
CP	S-RHH-AMF vs S-RHH	8.853	8.352–9.354	<0.001
CP	S-RHH-AMF vs S-CSH	12.023	11.522–12.524	<0.001
CP	S-RHH-AMF vs S	14.040	13.539–14.541	<0.001
CP	S-RHH-AMF vs CK	16.203	15.702–16.704	<0.001
CP	S-RHH vs S-CSH	3.170	2.669–3.671	<0.001

CP	S-RHH vs S	5.187	4.686–5.688	<0.001
CP	S-RHH vs CK	7.350	6.849–7.851	<0.001
CP	S-CSH vs S	2.017	1.516–2.518	<0.001
CP	S-CSH vs CK	4.180	3.679–4.681	<0.001
CP	S vs CK	2.163	1.662–2.664	<0.001

Note: Pairwise comparisons were obtained from the blocked treatment analyses for the 0–10 cm soil layer at Month 4, with treatment as the fixed factor and rhizobox identity included as the blocking factor. Mean differences are expressed in percentage points and correspond to the first treatment minus the second treatment. Confidence intervals and P values were adjusted for multiple comparisons among the five treatments using Tukey's procedure. Each treatment was represented by three treatment compartments, one in each rhizobox (n = 3). Sat.WC, saturated water content; FC, field capacity; TP, total porosity; CP, capillary porosity. P values below 0.001 are reported as $P < 0.001$.

Table S19. Estimated marginal means and Tukey groupings from the blocked analysis of alfalfa maximum root length.

Treatment	n	EMM (cm)	95% CI	SD (cm)	Tukey group
CK	3	14.47	13.75–15.20	0.75	e
S	3	16.67	15.95–17.40	0.63	d
S-CSH	3	22.52	21.79–23.25	0.75	c
S-RHH	3	25.68	24.95–26.41	1.10	b
S-RHH-AMF	3	33.46	32.74–34.19	1.01	a

Note: Values are estimated marginal means (EMMs) from a blocked ANOVA with treatment as the fixed factor and rhizobox identity included as the blocking factor. Each treatment was represented by three treatment compartments, one in each rhizobox (n = 3). SD values summarize the three observed treatment-compartment values within each treatment. Different lowercase letters indicate significant differences among treatments based on Tukey-adjusted comparisons ($P < 0.05$). The overall treatment effect was significant [$F(4, 8) = 576.44$, $P < 0.001$, partial $\eta^2 = 0.997$].

Table S20. Tukey-adjusted pairwise comparisons from the blocked analysis of alfalfa maximum root length.

comparison	Mean difference (cm)	95%CI	Tukey-adjusted P

S-RHH-AMF vs S-RHH	7.783	6.245–9.322	<0.001
S-RHH-AMF vs S-CSH	10.943	9.405–12.482	<0.001
S-RHH-AMF vs S	16.79	15.251–18.329	<0.001
S-RHH-AMF vs CK	18.99	17.451–20.529	<0.001
S-RHH vs S-CSH	3.16	1.621–4.699	<0.001
S-RHH vs S	9.007	7.468–10.545	<0.001
S-RHH vs CK	11.207	9.668–12.745	<0.001
S-CSH vs S	5.847	4.308–7.385	<0.001
S-CSH vs CK	8.047	6.508–9.585	<0.001
S vs CK	2.2	0.661–3.739	0.0073

Note: Pairwise treatment contrasts were obtained from the blocked ANOVA with treatment as the fixed factor and rhizobox identity included as the blocking factor. Mean differences are expressed as the first treatment minus the second treatment. Confidence intervals and P values were adjusted for multiple comparisons among the five treatments using Tukey's procedure. Each treatment was represented by three treatment compartments, one in each rhizobox (n = 3). P values below 0.001 are reported as P < 0.001.

Table S21. Total heavy metal concentrations in pot soil under different treatments (mg kg^{-1}).

Treatment	As	Cd	Cr	Cu	Hg	Ni	Pb	Zn
CK	2.431 ± 0.088	0.0907 ± 0.0041	16.144 ± 0.405	9.185 ± 0.363	0.0945 ± 0.0053	11.656 ± 0.416	23.901 ± 0.725	46.536 ± 2.414
CCH2	2.255 ± 0.058	0.0918 ± 0.0018	15.385 ± 0.483	8.987 ± 0.331	0.0824 ± 0.0046**	11.928 ± 0.404	21.931 ± 0.775*	42.074 ± 2.053*
CCH4	2.048 ± 0.036***	0.0848 ± 0.0009	15.331 ± 0.465	8.205 ± 0.574*	0.0794 ± 0.0052***	10.975 ± 0.451	20.264 ± 0.856***	40.846 ± 2.124**
CCH6	1.940 ± 0.080***	0.0856 ± 0.0027	15.423 ± 0.597	8.416 ± 0.328	0.0768 ± 0.0050***	10.233 ± 0.372**	19.665 ± 0.884***	40.658 ± 0.233**
CCH8	1.901 ± 0.088***	0.0824 ± 0.0049	13.589 ± 0.505***	7.645 ± 0.464***	0.0758 ± 0.0024***	10.185 ± 0.660**	18.722 ± 0.780***	39.438 ± 2.789***
CSH2	2.190 ± 0.142*	0.0891 ± 0.0067	15.231 ± 0.685	8.729 ± 0.306	0.0857 ± 0.0029*	11.749 ± 0.488	21.564 ± 0.633**	43.298 ± 0.907
CSH4	2.031 ± 0.054***	0.0825 ± 0.0021	14.779 ± 0.885	8.253 ± 0.280*	0.0762 ± 0.0042***	10.898 ± 0.267	20.716 ± 0.650***	40.367 ± 1.703**

CSH6	1.988 ± 0.090***	0.0849 ± 0.0029	14.300 ± 0.361**	7.994 ± 0.439**	0.0719 ± 0.0021***	9.980 ± 0.309***	20.287 ± 0.311***	38.595 ± 1.194***
CSH8	1.934 ± 0.066***	0.0793 ± 0.0056**	13.394 ± 0.813***	7.639 ± 0.179***	0.0723 ± 0.0035***	9.720 ± 0.467***	19.170 ± 0.713***	37.366 ± 1.156***
RHH2	2.047 ± 0.106***	0.0840 ± 0.0032	14.825 ± 0.254	8.233 ± 0.206*	0.0753 ± 0.0015***	10.318 ± 0.160**	19.507 ± 0.988***	40.040 ± 1.853***
RHH4	1.937 ± 0.068***	0.0889 ± 0.0039	14.435 ± 0.557*	7.991 ± 0.197**	0.0691 ± 0.0028***	9.952 ± 0.659***	19.120 ± 0.669***	38.303 ± 1.780***
RHH6	1.852 ± 0.094***	0.0799 ± 0.0005*	14.025 ± 0.582**	7.381 ± 0.334***	0.0630 ± 0.0029***	10.196 ± 0.297**	17.755 ± 0.557***	35.832 ± 1.179***
RHH8	1.753 ± 0.010***	0.0803 ± 0.0036*	12.912 ± 0.714***	7.269 ± 0.299***	0.0645 ± 0.0007***	9.580 ± 0.536***	16.728 ± 0.706***	33.887 ± 0.915***

Note: Values are mean ± SD (n = 3). Asterisks indicate significant differences from CK based on Dunnett-adjusted comparisons: * $P < 0.05$, ** $P < 0.01$, *** $P < 0.001$. CCH, corncob hydrochar; CSH, corn straw hydrochar; RHH, rice husk hydrochar; numbers following hydrochar abbreviations indicate application rates (%).

Table S22. One-way ANOVA for total heavy metal concentrations in pot soil.

Element	df	F	P	Partial η²	Levene P
As	12, 26	14.589	<0.001	0.871	0.350
Cd	12, 26	3.728	0.002	0.632	0.309
Cr	12, 26	7.572	<0.001	0.778	0.862
Cu	12, 26	8.535	<0.001	0.798	0.863
Hg	12, 26	16.870	<0.001	0.886	0.359
Ni	12, 26	9.582	<0.001	0.816	0.671
Pb	12, 26	19.385	<0.001	0.900	0.947
Zn	12, 26	10.813	<0.001	0.833	0.210

Note: Treatment was the fixed factor with 13 levels. Levene's tests were non-significant for all elements; therefore, conventional one-way ANOVA was retained. Partial η^2 represents the treatment effect size.

Table S23. Factorial ANOVA of hydrochar type and application rate effects on total heavy metal concentrations in pot soil.

Element	Effect	df	F	P	Partial η²
As	Hydrochar type	2, 24	11.654	<0.001	0.493
As	Application rate	3, 24	23.135	<0.001	0.743
As	Type × rate	6, 24	0.506	0.798	0.112
Cd	Hydrochar type	2, 24	2.001	0.157	0.143
Cd	Application rate	3, 24	6.855	0.002	0.462
Cd	Type × rate	6, 24	2.164	0.083	0.351
Cr	Hydrochar type	2, 24	6.533	0.005	0.353

Cr	Application rate	3, 24	16.520	<0.001	0.674
Cr	Type × rate	6, 24	0.480	0.817	0.107
Cu	Hydrochar type	2, 24	9.473	<0.001	0.441
Cu	Application rate	3, 24	16.643	<0.001	0.675
Cu	Type × rate	6, 24	0.871	0.531	0.179
Hg	Hydrochar type	2, 24	32.824	<0.001	0.732
Hg	Application rate	3, 24	18.672	<0.001	0.700
Hg	Type × rate	6, 24	0.978	0.462	0.196
Ni	Hydrochar type	2, 24	10.602	<0.001	0.469
Ni	Application rate	3, 24	19.281	<0.001	0.707
Ni	Type × rate	6, 24	2.576	0.045	0.392
Pb	Hydrochar type	2, 24	30.918	<0.001	0.720
Pb	Application rate	3, 24	23.806	<0.001	0.749
Pb	Type × rate	6, 24	0.624	0.709	0.135
Zn	Hydrochar type	2, 24	17.357	<0.001	0.591
Zn	Application rate	3, 24	14.899	<0.001	0.651
Zn	Type × rate	6, 24	1.091	0.396	0.214

Note: CK was excluded from the factorial analysis. The model included three hydrochar types (CCH, CSH, and RHH) and four application rates (2%, 4%, 6%, and 8%).

Table S24. Follow-up simple-effects analysis for Ni in pot soil.

Simple-effect omnibus tests

Simple effect	Level	F	df	P
Application rate within hydrochar type	CCH	8.499	3, 8	0.007
Application rate within hydrochar type	CSH	16.496	3, 8	<0.001
Application rate within hydrochar type	RHH	1.521	3, 8	0.282
Hydrochar type within application rate	2%	16.404	2, 6	0.004
Hydrochar type within application rate	4%	4.122	2, 6	0.075
Hydrochar type within application rate	6%	0.523	2, 6	0.618
Hydrochar type within application rate	8%	0.958	2, 6	0.436

Significant Tukey-adjusted follow-up comparisons

Simple effect	Level	Comparison	Mean difference (mg kg^{-1})	95% CI	Adjusted P
Rate within CCH	CCH	2% vs 6%	−1.695	−2.963 to −0.427	0.0115
Rate within CCH	CCH	2% vs 8%	−1.743	−3.011 to −0.475	0.0098
Rate within CSH	CSH	2% vs 6%	−1.769	−2.801 to −0.738	0.0026
Rate within CSH	CSH	2% vs 8%	−2.029	−3.061 to −0.998	0.0011
Rate within CSH	CSH	4% vs 8%	−1.179	−2.210 to −0.147	0.0265
Type within 2%	2%	CCH vs RHH	−1.609	−2.554 to −0.664	0.0047
Type within 2%	2%	CSH vs RHH	−1.430	−2.375 to −0.486	0.0084

Note: Simple-effects analyses were performed because the hydrochar type × application rate

interaction was significant for Ni (Table S23). In the pairwise comparisons, mean differences are expressed as the second level minus the first level. Only significant Tukey-adjusted pairwise comparisons are shown; all unlisted pairwise comparisons were non-significant (P > 0.05).

Table S25. Blocked mixed-design repeated-measures ANOVA for total heavy metal concentrations in rhizobox soil.

Element	Effect	df	F	P	Partial η²
As	Treatment	4, 8	501.329	<0.001	0.996
As	Soil layer	2, 20	2.921	0.077	0.226
As	Treatment × Soil layer	8, 20	1.759	0.146	0.413
Cd	Treatment	4, 8	22.880	<0.001	0.920
Cd	Soil layer	2, 20	2.955	0.075	0.228
Cd	Treatment × Soil layer	8, 20	1.364	0.271	0.353
Cr	Treatment	4, 8	72.515	<0.001	0.973
Cr	Soil layer	2, 20	5.142	0.016	0.340
Cr	Treatment × Soil layer	8, 20	1.216	0.339	0.327
Cu	Treatment	4, 8	116.782	<0.001	0.983
Cu	Soil layer	2, 20	0.442	0.649	0.042
Cu	Treatment × Soil layer	8, 20	0.262	0.971	0.095
Hg	Treatment	4, 8	333.211	<0.001	0.994
Hg	Soil layer	2, 20	0.235	0.793	0.023
Hg	Treatment × Soil layer	8, 20	0.187	0.990	0.070
Ni	Treatment	4, 8	131.941	<0.001	0.985
Ni	Soil layer	2, 20	0.529	0.597	0.050
Ni	Treatment × Soil layer	8, 20	1.180	0.359	0.321
Pb	Treatment	4, 8	221.160	<0.001	0.991
Pb	Soil layer	2, 20	1.224	0.315	0.109
Pb	Treatment × Soil layer	8, 20	0.706	0.684	0.220
Zn	Treatment	4, 8	399.463	<0.001	0.994
Zn	Soil layer	2, 20	3.821	0.039	0.276
Zn	Treatment × Soil layer	8, 20	1.494	0.221	0.374

Note: The treatment-specific compartment was the experimental unit, with each treatment represented by three compartments, one in each rhizobox (n = 3). Rhizobox identity was included as a blocking factor. Treatment was the fixed between-compartment factor, whereas soil layer (0 – 10, 10 – 20, and 20 – 30 cm) was the repeated within-compartment factor. Soil heavy metal concentrations were measured only at Month 4; therefore, sampling time was not included in this analysis. The treatment effect was evaluated using the between-compartment error after accounting for rhizobox blocking, whereas soil-layer and treatment × soil-layer effects were evaluated using the corresponding repeated-measures error stratum. Partial η^2 is reported as the effect size.

Table S26. Estimated marginal means of total heavy metal concentrations across soil layers from

the blocked repeated-measures analysis of the rhizobox experiment.

Element	Treatment	n	EMM (mg kg⁻¹)	95% CI
As	CK	3	0.634	0.575–0.694
As	S	3	2.086	2.026–2.145
As	S-CSH	3	1.911	1.851–1.971
As	S-RHH	3	1.847	1.787–1.906
As	S-RHH-AMF	3	1.798	1.738–1.858
Cd	CK	3	0.0586	0.0539–0.0632
Cd	S	3	0.0847	0.0800–0.0893
Cd	S-CSH	3	0.0782	0.0736–0.0829
Cd	S-RHH	3	0.0742	0.0696–0.0789
Cd	S-RHH-AMF	3	0.0717	0.0670–0.0763
Cr	CK	3	9.890	9.273–10.507
Cr	S	3	15.791	15.174–16.408
Cr	S-CSH	3	14.699	14.082–15.316
Cr	S-RHH	3	14.379	13.762–14.996
Cr	S-RHH-AMF	3	14.422	13.805–15.039
Cu	CK	3	3.224	2.740–3.709
Cu	S	3	8.884	8.400–9.369
Cu	S-CSH	3	8.134	7.650–8.619
Cu	S-RHH	3	7.952	7.468–8.437
Cu	S-RHH-AMF	3	7.944	7.460–8.429
Hg	CK	3	0.0297	0.0266–0.0328
Hg	S	3	0.0896	0.0864–0.0927
Hg	S-CSH	3	0.0868	0.0837–0.0899
Hg	S-RHH	3	0.0777	0.0746–0.0808
Hg	S-RHH-AMF	3	0.0807	0.0776–0.0838
Ni	CK	3	5.634	5.167–6.101
Ni	S	3	11.632	11.165–12.099
Ni	S-CSH	3	10.468	10.001–10.935
Ni	S-RHH	3	10.330	9.863–10.797
Ni	S-RHH-AMF	3	10.333	9.866–10.800
Pb	CK	3	12.261	11.568–12.954
Pb	S	3	23.610	22.917–24.303
Pb	S-CSH	3	21.706	21.012–22.399
Pb	S-RHH	3	21.847	21.154–22.540
Pb	S-RHH-AMF	3	20.837	20.144–21.530
Zn	CK	3	18.513	17.172–19.854
Zn	S	3	45.503	44.162–46.844
Zn	S-CSH	3	41.878	40.537–43.219
Zn	S-RHH	3	40.800	39.459–42.141
Zn	S-RHH-AMF	3	39.701	38.360–41.042

Note: EMM, estimated marginal mean. EMMs and 95% confidence intervals were obtained from the

blocked repeated-measures model and averaged across the three soil layers. Treatment was the fixed between-compartment factor, soil layer was the repeated within-compartment factor, and rhizobox identity was included as the blocking factor. Each treatment was represented by three treatment compartments, one in each rhizobox (n = 3).

Table S27. Tukey-adjusted comparisons of amendment treatments with S for rhizobox soil heavy metal concentrations.

Element	Comparison	Mean difference (mg kg⁻¹)	Change vs S (%)	95% CI	Adjusted P
As	S-CSH vs S	−0.174	−8.4	−0.301 to −0.048	0.0092
As	S-RHH vs S	−0.239	−11.5	−0.366 to −0.112	0.0013
As	S-RHH-AMF vs S	−0.288	−13.8	−0.415 to −0.161	<0.001
Cd	S-CSH vs S	−0.0064	−7.6	−0.0163 to 0.0034	0.2522
Cd	S-RHH vs S	−0.0104	−12.3	−0.0203 to −0.0006	0.0383
Cd	S-RHH-AMF vs S	−0.0130	−15.4	−0.0229 to −0.0031	0.0119
Cr	S-CSH vs S	−1.092	−6.9	−2.400 to 0.215	0.1088
Cr	S-RHH vs S	−1.412	−8.9	−2.720 to −0.105	0.0344
Cr	S-RHH-AMF vs S	−1.369	−8.7	−2.676 to −0.061	0.0401
Cu	S-CSH vs S	−0.750	−8.4	−1.777 to 0.277	0.1774
Cu	S-RHH vs S	−0.932	−10.5	−1.959 to 0.095	0.0772
Cu	S-RHH-AMF vs S	−0.940	−10.6	−1.967 to 0.087	0.0745
Hg	S-CSH vs S	−0.0028	−3.1	−0.0094 to 0.0038	0.6129
Hg	S-RHH vs S	−0.0119	−13.3	−0.0185 to −0.0053	0.0017
Hg	S-RHH-AMF vs S	−0.0089	−9.9	−0.0155 to −0.0023	0.0103
Ni	S-CSH vs S	−1.164	−10.0	−2.154 to −0.175	0.0221
Ni	S-RHH vs S	−1.302	−11.2	−2.292 to −0.313	0.0119

Ni	S-RHH-AMF vs S	−1.299	−11.2	−2.288 to −0.309	0.0121
Pb	S-CSH vs S	−1.904	−8.1	−3.373 to −0.436	0.0129
Pb	S-RHH vs S	−1.763	−7.5	−3.232 to −0.295	0.0198
Pb	S-RHH-AMF vs S	−2.773	−11.7	−4.242 to −1.305	0.0012
Zn	S-CSH vs S	−3.626	−8.0	−6.467 to −0.784	0.0142
Zn	S-RHH vs S	−4.703	−10.3	−7.545 to −1.862	0.0029
Zn	S-RHH-AMF vs S	−5.802	−12.8	−8.643 to −2.961	<0.001

Note: Comparisons were based on treatment estimated marginal means averaged across the three soil layers from the blocked repeated-measures analysis, with treatment as the fixed between-compartment factor and rhizobox identity included as the blocking factor. Mean differences are expressed as the amendment treatment minus S; negative values indicate lower total heavy metal concentrations than S. Confidence intervals and P values were adjusted for multiple comparisons among treatments using Tukey’ s procedure. Each treatment was represented by three treatment compartments, one in each rhizobox (n = 3). P values below 0.001 are reported as $P < 0.001$.

Table S28. Follow-up analysis of significant soil-layer effects for Cr and Zn in the rhizobox experiment.

Element	Result type	Layer/comparison	EMM or difference (mg kg^{-1})	95% CI	Holm-adjusted P
Cr	Marginal mean	0–10 cm	14.123	13.809–14.436	—
Cr	Marginal mean	10–20 cm	13.927	13.613–14.240	—
Cr	Marginal mean	20–30 cm	13.459	13.146–13.773	—
Cr	Pairwise	0–10 vs 10–20 cm	0.196	−0.247 to 0.639	0.367
Cr	Pairwise	0–10 vs 20–30 cm	0.663	0.220–1.107	**0.016**
Cr	Pairwise	10–20 vs 20–30 cm	0.467	0.024–0.911	0.080
Zn	Marginal mean	0–10 cm	37.839	36.946–38.733	—
Zn	Marginal mean	10–20 cm	37.681	36.788–38.575	—
Zn	Marginal mean	20–30 cm	36.317	35.423–37.210	—
Zn	Pairwise	0–10 vs 10–20 cm	0.158	−1.105 to 1.421	0.797
Zn	Pairwise	0–10 vs 20–30 cm	1.523	0.259–2.786	0.062
Zn	Pairwise	10–20 vs 20–30 cm	1.365	0.101–2.628	0.071

Note: Follow-up comparisons were conducted for Cr and Zn because their soil-layer main effects were significant in the blocked repeated-measures analysis reported in Table S25. Pairwise P values were

adjusted using the Holm procedure.

Table S29. Heavy metal concentrations (mg kg^{-1}) in shoots and roots of ryegrass under different treatments in the pot experiment.

Treatment	As	Cd	Cr	Cu	Hg	Ni	Pb	Zn
CK	0.2522 ± 0.0108 / 0.3270 ± 0.0300	0.0211 ± 0.0014 / 0.0282 ± 0.0038	0.6527 ± 0.0320 / 0.6850 ± 0.0400	0.3118 ± 0.0133 / 0.4140 ± 0.0300	0.4160 ± 0.0315 / 0.4350 ± 0.0230	0.6527 ± 0.0320 / 1.1283 ± 0.0828	0.5470 ± 0.0255 / 0.8873 ± 0.0635	0.7603 ± 0.0385 / 1.6850 ± 0.1350
CCH2	0.0985 ± 0.0071 / 0.1848 ± 0.0165	0.0018 ± 0.0002 / 0.0118 ± 0.0017	0.1737 ± 0.0117 / 0.3826 ± 0.0307	0.0836 ± 0.0059 / 0.2856 ± 0.0164	0.2348 ± 0.0307 / 0.2453 ± 0.0303	0.4716 ± 0.0300 / 0.9380 ± 0.0471	0.2363 ± 0.0149 / 0.5713 ± 0.0305	0.4219 ± 0.0266 / 1.0974 ± 0.0561
CCH4	0.1228 ± 0.0072 / 0.1918 ± 0.0166	0.0037 ± 0.0003 / 0.0126 ± 0.0015	0.2292 ± 0.0163 / 0.3984 ± 0.0271	0.1271 ± 0.0085 / 0.2928 ± 0.0190	0.2397 ± 0.0185 / 0.2955 ± 0.0205	0.5014 ± 0.0300 / 0.9616 ± 0.0500	0.2940 ± 0.0186 / 0.6021 ± 0.0317	0.5033 ± 0.0320 / 1.1344 ± 0.0600
CCH6	0.1479 ± 0.0092 / 0.2010 ± 0.0166	0.0042 ± 0.0003 / 0.0131 ± 0.0014	0.2623 ± 0.0167 / 0.4147 ± 0.0299	0.1561 ± 0.0102 / 0.3208 ± 0.0200	0.2617 ± 0.0202 / 0.3143 ± 0.0215	0.5988 ± 0.0329 / 0.9728 ± 0.0520	0.3258 ± 0.0199 / 0.6465 ± 0.0344	0.5572 ± 0.0349 / 1.2890 ± 0.0644
CCH8	0.1686 ± 0.0100 / 0.2069 ± 0.0167	0.0057 ± 0.0004 / 0.0147 ± 0.0015	0.2935 ± 0.0190 / 0.4283 ± 0.0300	0.1589 ± 0.0100 / 0.3493 ± 0.0214	0.2920 ± 0.0207 / 0.3304 ± 0.0214	0.6377 ± 0.0351 / 0.9961 ± 0.0556	0.3895 ± 0.0236 / 0.6805 ± 0.0351	0.6540 ± 0.0379 / 1.3533 ± 0.0681
CSH2	0.1227 ± 0.0072 / 0.1262 ± 0.0120	0.0057 ± 0.0004 / 0.0084 ± 0.0012	0.3784 ± 0.0240 / 0.3319 ± 0.0266	0.1468 ± 0.0102 / 0.3150 ± 0.0200	0.2397 ± 0.0187 / 0.2704 ± 0.0187	0.7294 ± 0.0429 / 0.7954 ± 0.0450	0.3753 ± 0.0237 / 0.6365 ± 0.0344	0.6493 ± 0.0365 / 1.1122 ± 0.0621

CSH4	0.1305 ± 0.0080 / 0.1300 ± 0.0120	0.0088 ± 0.0007 / 0.0098 ± 0.0014	0.4106 ± 0.0249 / 0.3982 ± 0.0271	0.2175 ± 0.0144 / 0.3959 ± 0.0252	0.2683 ± 0.0200 / 0.2920 ± 0.0205	0.7469 ± 0.0450 / 0.9463 ± 0.0516	0.4255 ± 0.0264 / 0.6815 ± 0.0335	0.8491 ± 0.0471 / 1.7581 ± 0.0834
CSH6	0.1430 ± 0.0084 / 0.1419 ± 0.0129	0.0104 ± 0.0007 / 0.0116 ± 0.0016	0.4572 ± 0.0285 / 0.4550 ± 0.0302	0.3038 ± 0.0184 / 0.4292 ± 0.0287	0.2927 ± 0.0217 / 0.3173 ± 0.0216	0.7714 ± 0.0450 / 1.0781 ± 0.0570	0.5001 ± 0.0314 / 0.7366 ± 0.0344	1.0867 ± 0.0599 / 1.9153 ± 0.0955
CSH8	0.1535 ± 0.0090 / 0.1595 ± 0.0146	0.0115 ± 0.0009 / 0.0133 ± 0.0015	0.4841 ± 0.0292 / 0.5241 ± 0.0337	0.3743 ± 0.0222 / 0.5027 ± 0.0321	0.3237 ± 0.0221 / 0.3251 ± 0.0237	0.7854 ± 0.0464 / 1.1426 ± 0.0581	0.5605 ± 0.0350 / 0.7804 ± 0.0351	1.2549 ± 0.0681 / 2.1575 ± 0.1067
RHH2	0.1149 ± 0.0065 / 0.1276 ± 0.0126	0.0021 ± 0.0002 / 0.0028 ± 0.0004	0.1737 ± 0.0115 / 0.2476 ± 0.0235	0.0688 ± 0.0047 / 0.1100 ± 0.0115	0.2140 ± 0.0150 / 0.2586 ± 0.0200	0.5001 ± 0.0314 / 0.8255 ± 0.0450	0.2756 ± 0.0171 / 0.6589 ± 0.0371	0.2393 ± 0.0149 / 0.4853 ± 0.0270
RHH4	0.1334 ± 0.0079 / 0.1908 ± 0.0170	0.0037 ± 0.0003 / 0.0084 ± 0.0012	0.1936 ± 0.0120 / 0.3857 ± 0.0352	0.1026 ± 0.0072 / 0.2980 ± 0.0235	0.2340 ± 0.0150 / 0.2730 ± 0.0221	0.5105 ± 0.0314 / 0.8982 ± 0.0471	0.3255 ± 0.0199 / 0.7315 ± 0.0335	0.3691 ± 0.0237 / 0.8160 ± 0.0379
RHH6	0.1346 ± 0.0080 / 0.2175 ± 0.0201	0.0045 ± 0.0004 / 0.0121 ± 0.0017	0.2159 ± 0.0130 / 0.4054 ± 0.0357	0.1354 ± 0.0099 / 0.3755 ± 0.0270	0.2529 ± 0.0166 / 0.2981 ± 0.0231	0.5261 ± 0.0337 / 0.9046 ± 0.0464	0.3752 ± 0.0237 / 0.7713 ± 0.0335	0.4846 ± 0.0292 / 1.1639 ± 0.0510
RHH8	0.1411 ± 0.0087 / 0.2596 ± 0.0250	0.0054 ± 0.0005 / 0.0161 ± 0.0020	0.2388 ± 0.0140 / 0.4423 ± 0.0381	0.1492 ± 0.0107 / 0.4741 ± 0.0337	0.2663 ± 0.0171 / 0.3271 ± 0.0250	0.5566 ± 0.0349 / 0.9453 ± 0.0500	0.4178 ± 0.0244 / 0.8193 ± 0.0344	0.5211 ± 0.0314 / 1.7892 ± 0.0644

Note: Values are means ± SD (n = 3). Within each cell, shoot and root concentrations are presented as shoot / root.

Table S30. Effects of treatment, plant organ, and their interaction on heavy metal concentrations in ryegrass.

Panel A. Overall mixed-design repeated-measures ANOVA

Metal	Effect	F (df)	P	partial η²
As	Treatment	237.79 (12, 26)	<0.001	0.991
As	Organ	122.61 (1, 26)	<0.001	0.825
As	Treatment × Organ	6.89 (12, 26)	<0.001	0.761
Cd	Treatment	226.17 (12, 26)	<0.001	0.991
Cd	Organ	239.74 (1, 26)	<0.001	0.902
Cd	Treatment × Organ	8.19 (12, 26)	<0.001	0.791
Cr	Treatment	807.28 (12, 26)	<0.001	0.997
Cr	Organ	154.64 (1, 26)	<0.001	0.856
Cr	Treatment × Organ	9.50 (12, 26)	<0.001	0.814
Cu	Treatment	570.96 (12, 26)	<0.001	0.996
Cu	Organ	865.71 (1, 26)	<0.001	0.971
Cu	Treatment × Organ	10.59 (12, 26)	<0.001	0.830
Hg	Treatment	1387.87 (12, 26)	<0.001	0.998
Hg	Organ	24.26 (1, 26)	<0.001	0.483
Hg	Treatment × Organ	0.51 (12, 26)	0.889	0.191
Ni	Treatment	258.69 (12, 26)	<0.001	0.992
Ni	Organ	590.55 (1, 26)	<0.001	0.958
Ni	Treatment × Organ	4.70 (12, 26)	<0.001	0.684
Pb	Treatment	400.84 (12, 26)	<0.001	0.995
Pb	Organ	1089.17 (1, 26)	<0.001	0.977
Pb	Treatment × Organ	3.42 (12, 26)	0.004	0.612
Zn	Treatment	1090.87 (12, 26)	<0.001	0.998
Zn	Organ	1569.77 (1, 26)	<0.001	0.984
Zn	Treatment × Organ	15.39 (12, 26)	<0.001	0.877

Panel B. Simple effects of treatment within each organ

Metal	Organ	F (df)	P	partial η²
As	Shoot	59.22 (12, 26)	<0.001	0.965
As	Root	31.02 (12, 26)	<0.001	0.935
Cd	Shoot	227.83 (12, 26)	<0.001	0.991
Cd	Root	33.27 (12, 26)	<0.001	0.939
Cr	Shoot	149.91 (12, 26)	<0.001	0.986
Cr	Root	30.83 (12, 26)	<0.001	0.934
Cu	Shoot	182.46 (12, 26)	<0.001	0.988
Cu	Root	50.52 (12, 26)	<0.001	0.959
Hg	Shoot	18.41 (12, 26)	<0.001	0.895
Hg	Root	13.20 (12, 26)	<0.001	0.859
Ni	Shoot	28.79 (12, 26)	<0.001	0.930

Ni	Root	11.23 (12, 26)	<0.001	0.838
Pb	Shoot	53.28 (12, 26)	<0.001	0.961
Pb	Root	17.77 (12, 26)	<0.001	0.891
Zn	Shoot	157.37 (12, 26)	<0.001	0.986
Zn	Root	117.65 (12, 26)	<0.001	0.982

Note: Treatment was the between-subject factor and organ (shoot/root) was the within-subject factor; individual pots were experimental subjects.

Table S31. Dunnett-adjusted comparisons of heavy metal concentrations in ryegrass shoots and roots between hydrochar treatments and the control in the pot experiment.

Panel A. Shoots

Treatment	As	Cd	Cr	Cu	Hg	Ni	Pb	Zn
CCH2	−0.1537 (<0.001)	−0.0193 (<0.001)	−0.4790 (<0.001)	−0.2283 (<0.001)	−0.1812 (<0.001)	−0.1810 (<0.001)	−0.3107 (<0.001)	−0.3384 (<0.001)
CCH4	−0.1294 (<0.001)	−0.0174 (<0.001)	−0.4235 (<0.001)	−0.1848 (<0.001)	−0.1763 (<0.001)	−0.1512 (<0.001)	−0.2530 (<0.001)	−0.2570 (<0.001)
CCH6	−0.1043 (<0.001)	−0.0169 (<0.001)	−0.3903 (<0.001)	−0.1557 (<0.001)	−0.1543 (<0.001)	−0.0538 (0.450)	−0.2212 (<0.001)	−0.2031 (<0.001)
CCH8	−0.0836 (<0.001)	−0.0154 (<0.001)	−0.3592 (<0.001)	−0.1529 (<0.001)	−0.1240 (<0.001)	−0.0150 (1.000)	−0.1575 (<0.001)	−0.1064 (0.024)
CSH2	−0.1295 (<0.001)	−0.0154 (<0.001)	−0.2742 (<0.001)	−0.1650 (<0.001)	−0.1763 (<0.001)	+0.0767 (0.120)	−0.1717 (<0.001)	−0.1111 (0.017)
CSH4	−0.1217 (<0.001)	−0.0123 (<0.001)	−0.2421 (<0.001)	−0.0944 (<0.001)	−0.1477 (<0.001)	+0.0942 (0.034)	−0.1215 (<0.001)	+0.0888 (0.081)
CSH6	−0.1091 (<0.001)	−0.0107 (<0.001)	−0.1955 (<0.001)	−0.0081 (0.985)	−0.1233 (<0.001)	+0.1188 (0.005)	−0.0469 (0.166)	+0.3263 (<0.001)
CSH8	−0.0986 (<0.001)	−0.0095 (<0.001)	−0.1686 (<0.001)	+0.0625 (<0.001)	−0.0923 (<0.001)	+0.1327 (0.002)	+0.0135 (0.996)	+0.4946 (<0.001)
RHH2	−0.1372 (<0.001)	−0.0190 (<0.001)	−0.4790 (<0.001)	−0.2430 (<0.001)	−0.2020 (<0.001)	−0.1526 (<0.001)	−0.2714 (<0.001)	−0.5210 (<0.001)
RHH4	−0.1188 (<0.001)	−0.0174 (<0.001)	−0.4591 (<0.001)	−0.2092 (<0.001)	−0.1820 (<0.001)	−0.1421 (<0.001)	−0.2215 (<0.001)	−0.3912 (<0.001)
RHH6	−0.1176 (<0.001)	−0.0166 (<0.001)	−0.4367 (<0.001)	−0.1764 (<0.001)	−0.1631 (<0.001)	−0.1266 (0.003)	−0.1718 (<0.001)	−0.2758 (<0.001)
RHH8	−0.1111 (<0.001)	−0.0157 (<0.001)	−0.4139 (<0.001)	−0.1627 (<0.001)	−0.1497 (<0.001)	−0.0960 (0.030)	−0.1292 (<0.001)	−0.2392 (<0.001)

Panel B. Roots

Treatment	As	Cd	Cr	Cu	Hg	Ni	Pb	Zn
CCH2	−0.1422 (<0.001)	−0.0163 (<0.001)	−0.3024 (<0.001)	−0.1284 (<0.001)	−0.1897 (<0.001)	−0.1903 (0.002)	−0.3161 (<0.001)	−0.5876 (<0.001)
CCH4	−0.1352 (<0.001)	−0.0156 (<0.001)	−0.2866 (<0.001)	−0.1212 (<0.001)	−0.1395 (<0.001)	−0.1667 (0.007)	−0.2852 (<0.001)	−0.5506 (<0.001)

CCH6	−0.1260 (<0.001)	−0.0151 (<0.001)	−0.2703 (<0.001)	−0.0932 (<0.001)	−0.1207 (<0.001)	−0.1556 (0.014)	−0.2408 (<0.001)	−0.3960 (<0.001)
CCH8	−0.1201 (<0.001)	−0.0135 (<0.001)	−0.2567 (<0.001)	−0.0647 (0.029)	−0.1046 (<0.001)	−0.1323 (0.046)	−0.2068 (<0.001)	−0.3317 (<0.001)
CSH2	−0.2008 (<0.001)	−0.0198 (<0.001)	−0.3531 (<0.001)	−0.0990 (<0.001)	−0.1646 (<0.001)	−0.3329 (<0.001)	−0.2509 (<0.001)	−0.5728 (<0.001)
CSH4	−0.1970 (<0.001)	−0.0183 (<0.001)	−0.2868 (<0.001)	−0.0181 (0.970)	−0.1430 (<0.001)	−0.1820 (0.003)	−0.2059 (<0.001)	+0.0731 (0.859)
CSH6	−0.1851 (<0.001)	−0.0166 (<0.001)	−0.2300 (<0.001)	+0.0152 (0.991)	−0.1177 (<0.001)	−0.0502 (0.882)	−0.1508 (<0.001)	+0.2303 (0.008)
CSH8	−0.1675 (<0.001)	−0.0149 (<0.001)	−0.1609 (<0.001)	+0.0887 (0.001)	−0.1099 (<0.001)	+0.0143 (1.000)	−0.1070 (0.014)	+0.4725 (<0.001)
RHH2	−0.1994 (<0.001)	−0.0254 (<0.001)	−0.4374 (<0.001)	−0.3040 (<0.001)	−0.1764 (<0.001)	−0.3029 (<0.001)	−0.2285 (<0.001)	−1.1997 (<0.001)
RHH4	−0.1362 (<0.001)	−0.0198 (<0.001)	−0.2993 (<0.001)	−0.1160 (<0.001)	−0.1620 (<0.001)	−0.2301 (<0.001)	−0.1559 (<0.001)	−0.8690 (<0.001)
RHH6	−0.1095 (<0.001)	−0.0160 (<0.001)	−0.2796 (<0.001)	−0.0385 (0.373)	−0.1369 (<0.001)	−0.2237 (<0.001)	−0.1160 (0.007)	−0.5211 (<0.001)
RHH8	−0.0674 (<0.001)	−0.0120 (<0.001)	−0.2427 (<0.001)	+0.0601 (0.047)	−0.1079 (<0.001)	−0.1831 (0.003)	−0.0680 (0.218)	+0.1042 (0.520)

Note: Values are mean differences between each hydrochar treatment and CK (treatment − CK, $mg\ kg^{-1}$), with Dunnett-adjusted P values in parentheses. Negative values indicate lower concentrations than CK and positive values indicate higher concentrations than CK. Comparisons were performed separately for shoots and roots. n = 3 independent pots per treatment. $P < 0.05$ was considered statistically significant.

Table S32. Organ-specific contrasts and effects of hydrochar type and application rate on heavy metal concentrations in ryegrass.

Panel A. Root − shoot contrasts within individual treatments

Treatment	As	Cd	Cr	Cu	Hg	Ni	Pb	Zn
CK	+0.0748 (0.605)	+0.0071 (0.692)	+0.0323 (1.000)	+0.1022 (0.175)	+0.0190 (1.000)	+0.4757 (0.147)	+0.3403 (0.080)	+0.9247 (0.061)
CCH2	+0.0863 (0.313)	+0.0101 (0.139)	+0.2089 (0.174)	+0.2020 (0.052)	+0.0105 (1.000)	+0.4664 (0.117)	+0.3350 (0.074)	+0.6755 (0.055)
CCH4	+0.0691 (0.397)	+0.0089 (0.151)	+0.1692 (0.233)	+0.1657 (0.086)	+0.0557 (1.000)	+0.4602 (0.119)	+0.3081 (0.074)	+0.6311 (0.061)
CCH6	+0.0531 (0.562)	+0.0089 (0.142)	+0.1524 (0.239)	+0.1647 (0.086)	+0.0526 (1.000)	+0.3739 (0.147)	+0.3207 (0.074)	+0.7318 (0.061)
CCH8	+0.0383 (0.785)	+0.0089 (0.151)	+0.1348 (0.289)	+0.1904 (0.086)	+0.0384 (1.000)	+0.3584 (0.147)	+0.2910 (0.080)	+0.6993 (0.061)
CSH2	+0.0036 (1.000)	+0.0026 (0.591)	−0.0465 (1.000)	+0.1682 (0.086)	+0.0308 (1.000)	+0.0660 (0.323)	+0.2612 (0.080)	+0.4629 (0.061)

CSH4	−0.0005 (1.000)	+0.0010 (1.000)	−0.0124 (1.000)	+0.1785 (0.086)	+0.0237 (1.000)	+0.1994 (0.147)	+0.2559 (0.080)	+0.9090 (0.061)
CSH6	−0.0011 (1.000)	+0.0012 (1.000)	−0.0022 (1.000)	+0.1255 (0.175)	+0.0246 (1.000)	+0.3067 (0.147)	+0.2364 (0.080)	+0.8287 (0.061)
CSH8	+0.0060 (1.000)	+0.0017 (1.000)	+0.0400 (1.000)	+0.1284 (0.175)	+0.0014 (1.000)	+0.3572 (0.147)	+0.2199 (0.080)	+0.9026 (0.061)
RHH2	+0.0127 (1.000)	+0.0007 (0.692)	+0.0739 (0.403)	+0.0412 (0.175)	+0.0446 (1.000)	+0.3254 (0.147)	+0.3833 (0.074)	+0.2459 (0.061)
RHH4	+0.0574 (0.517)	+0.0047 (0.219)	+0.1922 (0.233)	+0.1954 (0.086)	+0.0390 (1.000)	+0.3877 (0.147)	+0.4060 (0.074)	+0.4468 (0.061)
RHH6	+0.0829 (0.397)	+0.0077 (0.178)	+0.1895 (0.233)	+0.2400 (0.086)	+0.0452 (1.000)	+0.3785 (0.147)	+0.3961 (0.074)	+0.6794 (0.055)
RHH8	+0.1185 (0.313)	+0.0107 (0.152)	+0.2035 (0.233)	+0.3249 (0.074)	+0.0609 (1.000)	+0.3886 (0.147)	+0.4015 (0.074)	+1.2681 (0.025)

Panel B. Factorial effects of hydrochar type and application rate

Metal	Organ	Hydrochar type, F (P; ηp^2)	Application rate, F (P; ηp^2)	Type × rate, F (P; ηp^2)
As	Shoot	1.87 (0.176; 0.135)	44.59 (<0.001; 0.848)	5.66 (<0.001; 0.586)
As	Root	49.99 (<0.001; 0.806)	23.06 (<0.001; 0.742)	7.20 (<0.001; 0.643)
Cd	Shoot	465.87 (<0.001; 0.975)	129.74 (<0.001; 0.942)	4.89 (0.002; 0.550)
Cd	Root	15.43 (<0.001; 0.563)	38.32 (<0.001; 0.827)	8.22 (<0.001; 0.673)
Cr	Shoot	475.78 (<0.001; 0.975)	41.97 (<0.001; 0.840)	1.39 (0.258; 0.258)
Cr	Root	10.43 (<0.001; 0.465)	35.03 (<0.001; 0.814)	4.97 (0.002; 0.554)
Cu	Shoot	531.67 (<0.001; 0.978)	196.51 (<0.001; 0.961)	30.07 (<0.001; 0.883)
Cu	Root	65.90 (<0.001; 0.846)	115.87 (<0.001; 0.935)	21.81 (<0.001; 0.845)
Hg	Shoot	11.69 (<0.001; 0.493)	17.32 (<0.001; 0.684)	0.54 (0.771; 0.119)
Hg	Root	0.86 (0.434; 0.067)	15.99 (<0.001; 0.667)	0.54 (0.775; 0.118)
Ni	Shoot	143.33 (<0.001; 0.923)	11.75 (<0.001; 0.595)	2.22 (0.076; 0.357)
Ni	Root	12.07 (<0.001; 0.501)	19.87 (<0.001; 0.713)	5.70 (<0.001; 0.588)
Pb	Shoot	136.26 (<0.001; 0.919)	74.91 (<0.001; 0.904)	0.88 (0.523; 0.181)
Pb	Root	39.41 (<0.001; 0.767)	27.53 (<0.001; 0.775)	0.37 (0.891; 0.085)
Zn	Shoot	649.20 (<0.001; 0.982)	151.72 (<0.001; 0.950)	16.41 (<0.001; 0.804)
Zn	Root	320.00 (<0.001; 0.964)	259.18 (<0.001; 0.970)	40.08 (<0.001; 0.909)

Note: In Panel A, values are root − shoot mean differences ($mg\ kg^{-1}$), with Holm-adjusted P values in parentheses; positive values indicate higher concentrations in roots. Holm adjustment was applied across the 13 treatment-specific organ contrasts within each element. In Panel B, CK was excluded and the 12 hydrochar treatments were analyzed as a 3 (hydrochar type: CSH, CCH, RHH) × 4 (application rate: 2%, 4%, 6%, 8%) factorial design separately for shoots and roots. Degrees of freedom were (2, 24) for hydrochar type, (3, 24) for application rate, and (6, 24) for type × rate. ηp^2 denotes partial eta squared. n = 3 independent pots per treatment.

Table S33. Heavy metal concentrations in alfalfa shoots and roots and their blocked treatment-

and organ-dependent responses in the rhizobox experiment.

Panel A. Heavy metal concentrations

Treatment	As	Cd	Cr	Cu	Hg	Ni	Pb	Zn
CK	0.1200 ± 0.0100 / 0.2667 ± 0.0513	0.0107 ± 0.0012 / 0.0293 ± 0.0012	0.4567 ± 0.0306 / 1.1100 ± 0.0100	1.2167 ± 0.0115 / 3.5267 ± 0.0208	0.0100 ± 0.0000 / 0.0133 ± 0.0058	0.8500 ± 0.0200 / 2.1100 ± 0.0100	0.6167 ± 0.0058 / 2.8233 ± 0.0115	3.2333 ± 0.0231 / 12.5400 ± 0.0265
S	0.3433 ± 0.0208 / 0.8533 ± 0.0252	0.0333 ± 0.0058/ 0.0750 ± 0.0050	1.2133 ± 0.0153 / 3.2000 ± 0.0100	3.8200 ± 0.0100 / 9.5400 ± 0.0200	0.0167 ± 0.0058 / 0.0233 ± 0.0058	2.0000 ± 0.0100 / 5.2100 ± 0.0100	1.8300 ± 0.0100 / 6.5333 ± 0.0252	10.5467 ± 0.0379 / 32.0500 ± 0.0346
S-CSH	0.3100 ± 0.0265 / 0.7733 ± 0.0208	0.0327 ± 0.0064 / 0.0733 ± 0.0058	1.1033 ± 0.0252 / 2.9533 ± 0.0351	3.5233 ± 0.0153 / 8.8433 ± 0.0115	0.0133 ± 0.0058 / 0.0227 ± 0.0064	1.8400 ± 0.0100 / 4.8200 ± 0.0265	1.6600 ± 0.0173 / 6.0433 ± 0.0416	9.8300 ± 0.0265 / 29.5333 ± 0.0153
S-RHH	0.2967 ± 0.0252 / 0.7367 ± 0.0153	0.0290 ± 0.0017 / 0.0657 ± 0.0051	1.0567 ± 0.0503 / 2.7967 ± 0.0153	3.4300 ± 0.0265 / 8.4400 ± 0.0265	0.0133 ± 0.0058 / 0.0200 ± 0.0000	1.7633 ± 0.0379 / 4.6100 ± 0.0100	1.5600 ± 0.0265 / 5.7533 ± 0.0351	9.4267 ± 0.0208 / 28.2433 ± 0.0321
S-RHH-AMF	0.2867 ± 0.0252 / 0.7233 ± 0.0153	0.0320 ± 0.0072 / 0.0717 ± 0.0076	1.0233 ± 0.0153 / 2.7233 ± 0.0306	3.3600 ± 0.0100 / 8.2267 ± 0.0208	0.0100 ± 0.0000 / 0.0200 ± 0.0000	1.7533 ± 0.0351 / 4.5200 ± 0.0173	1.5333 ± 0.0153 / 5.6100 ± 0.0200	9.2433 ± 0.0153 / 27.5267 ± 0.0115

Panel B. Blocked mixed-design repeated-measures ANOVA

Metal	Treatment, F(4,8)	Organ, F(1,10)	Treatment × organ, F(4,10)
As	2948.16 (<0.001; 0.999)	5695.25 (<0.001; 0.998)	148.63 (<0.001; 0.983)
Cd	59.72 (<0.001; 0.968)	1938.52 (<0.001; 0.995)	28.26 (<0.001; 0.919)
Cr	3760.24 (<0.001; 0.999)	18928.57 (<0.001; 0.999)	427.73 (<0.001; 0.994)
Cu	57558.67 (<0.001; 1.000)	882782.26 (<0.001; 1.000)	14821.03 (<0.001; 1.000)
Hg	4.67 (0.031; 0.700)	19.31 (0.001; 0.659)	0.52 (0.724; 0.172)
Ni	9064.66 (<0.001; 1.000)	125889.84 (<0.001; 1.000)	2213.04 (<0.001; 0.999)
Pb	13071.66 (<0.001; 1.000)	447339.75 (<0.001; 1.000)	5643.68 (<0.001; 1.000)
Zn	495846.09 (<0.001; 1.000)	12336583.14 (<0.001; 1.000)	181520.20 (<0.001; 1.000)

Panel C. Blocked simple effects of treatment within each organ

Metal	Shoot, F(4,8)	Root, F(4,8)
As	428.31 (<0.001; 0.995)	642.76 (<0.001; 0.997)
Cd	20.47 (<0.001; 0.911)	89.18 (<0.001; 0.978)
Cr	269.89 (<0.001; 0.993)	3334.91 (<0.001; 0.999)
Cu	14943.23 (<0.001; 1.000)	62926.80 (<0.001; 1.000)
Hg	1.00 (0.461; 0.333)	4.29 (0.038; 0.682)

Ni	886.78 (<0.001; 0.998)	31182.00 (<0.001; 1.000)
Pb	4315.64 (<0.001; 1.000)	13227.94 (<0.001; 1.000)
Zn	91293.03 (<0.001; 1.000)	628992.86 (<0.001; 1.000)

Note: In Panel A, values are means ± SD (n = 3 treatment compartments per treatment, one in each rhizobox) and are presented as shoot/root concentrations (mg kg^{-1}). In Panel B, treatment was the fixed between-compartment factor, organ (shoot/root) was the repeated within-compartment factor, and rhizobox identity was included as the blocking factor. Treatment effects were tested using the between-compartment error after accounting for rhizobox blocking [F(4,8)], whereas organ and treatment × organ effects were evaluated using the corresponding within-compartment error terms. Values in parentheses following each F statistic are P value and partial eta squared (ηp^2), respectively. Panel C presents blocked simple effects of treatment analyzed separately within shoots and roots [F(4,8)].

Table S34. Planned comparisons from blocked analyses of treatment and organ effects on heavy metal concentrations in alfalfa in the rhizobox experiment.

Panel A. Amendment treatments versus the S treatment

Shoots

Treatment	As	Cd	Cr	Cu	Hg	Ni	Pb	Zn
S-CSH	−0.0333 (0.001)	−0.0007 (0.992)	−0.1100 (0.007)	−0.2967 (<0.001)	−0.0033 (0.741)	−0.1600 (<0.001)	−0.1700 (<0.001)	−0.7167 (<0.001)
S-RHH	−0.0467 (<0.001)	−0.0043 (0.388)	−0.1567 (<0.001)	−0.3900 (<0.001)	−0.0033 (0.741)	−0.2367 (<0.001)	−0.2700 (<0.001)	−1.1200 (<0.001)
S-RHH-AMF	−0.0567 (<0.001)	−0.0013 (0.943)	−0.1900 (<0.001)	−0.4600 (<0.001)	−0.0067 (0.282)	−0.2467 (<0.001)	−0.2967 (<0.001)	−1.3033 (<0.001)

Roots

Treatment	As	Cd	Cr	Cu	Hg	Ni	Pb	Zn
S-CSH	−0.0800 (<0.001)	−0.0017 (0.888)	−0.2467 (<0.001)	−0.6967 (<0.001)	−0.0007 (0.989)	−0.3900 (<0.001)	−0.4900 (<0.001)	−2.5167 (<0.001)
S-RHH	−0.1167 (<0.001)	−0.0093 (0.029)	−0.4033 (<0.001)	−1.1000 (<0.001)	−0.0033 (0.501)	−0.6000 (<0.001)	−0.7800 (<0.001)	−3.8067 (<0.001)
S-RHH-AMF	−0.1300 (<0.001)	−0.0033 (0.544)	−0.4767 (<0.001)	−1.3133 (<0.001)	−0.0033 (0.501)	−0.6900 (<0.001)	−0.9233 (<0.001)	−4.5233 (<0.001)

Panel B. Additional effect of AMF: S-RHH-AMF versus S-RHH

Organ	As	Cd	Cr	Cu	Hg	Ni	Pb	Zn
Shoot	−0.0100 (0.660)	+0.0030 (1.000)	−0.0333 (0.910)	−0.0700 (0.003)	−0.0033 (1.000)	−0.0100 (1.000)	−0.0267 (0.187)	−0.1833 (<0.001)
Root	−0.0133	+0.0060	−0.0733	−0.2133	0.0000	−0.0900	−0.1433	−0.7167

	(0.664)	(0.209)	(0.027)	(<0.001)	(1.000)	(<0.001)	(<0.001)	(<0.001)

Panel C. Root − shoot contrasts within individual treatments

Treatment	As	Cd	Cr	Cu	Hg	Ni	Pb	Zn
CK	+0.1467 (0.026)	+0.0187 (0.010)	+0.6533 (0.001)	+2.3100 (<0.001)	+0.0033 (0.734)	+1.2600 (<0.001)	+2.2067 (<0.001)	+9.3067 (<0.001)
S	+0.5100 (<0.001)	+0.0417 (0.005)	+1.9867 (<0.001)	+5.7200 (<0.001)	+0.0067 (0.734)	+3.2100 (<0.001)	+4.7033 (<0.001)	+21.5033 (<0.001)
S-CSH	+0.4633 (<0.001)	+0.0407 (0.001)	+1.8500 (0.001)	+5.3200 (<0.001)	+0.0093 (0.734)	+2.9800 (<0.001)	+4.3833 (<0.001)	+19.7033 (<0.001)
S-RHH	+0.4400 (<0.001)	+0.0367 (0.010)	+1.7400 (0.001)	+5.0100 (<0.001)	+0.0067 (0.734)	+2.8467 (<0.001)	+4.1933 (<0.001)	+18.8167 (<0.001)
S-RHH-AMF	+0.4367 (<0.001)	+0.0397 (<0.001)	+1.7000 (<0.001)	+4.8667 (<0.001)	+0.0100 (<0.001)	+2.7667 (<0.001)	+4.0767 (<0.001)	+18.2833 (<0.001)

Note: In Panel A, values are mean differences between each amendment treatment and S (treatment − S, mg kg^{-1}), with Dunnett-adjusted P values in parentheses; negative values indicate lower concentrations than S. Comparisons were obtained separately for shoots and roots from blocked treatment models, with treatment as the fixed factor and rhizobox identity included as the blocking factor. Dunnett adjustment was applied to the three amendment-versus-S contrasts within each metal and organ. In Panel B, values represent S-RHH-AMF − S-RHH mean differences (mg kg^{-1}), with Holm-adjusted P values in parentheses; negative values indicate lower concentrations following AMF inoculation. Holm adjustment was applied across the eight metals separately within each organ. In Panel C, values are paired root − shoot mean differences (mg kg^{-1}) within the same treatment compartments, with Holm-adjusted P values in parentheses; positive values indicate higher concentrations in roots. Holm adjustment was applied across the five treatment-specific organ contrasts within each element. Each treatment was represented by three treatment compartments, one in each rhizobox (n = 3). P < 0.05 was considered statistically significant.

Table S35. Summary of major soil and plant responses across treatments in the pot and rhizobox experiments.

Panel A. Pot experiment with ryegrass

Treatment	Major soil responses	Ryegrass growth and root responses	Plant metal accumulation	Key statistical evidence
CK	Reference; pH and EC decreased after cultivation	Reference	Reference	Significant time effects for pH and EC
CCH2	↑ AP, AK and SOM; AH-N ≈ CK	↑ growth and root traits	↓ selected metals	AP, AK and SOM > CK; AH-N ≈ CK

CCH4	↑ AP, AK, AH-N and SOM	↑ growth and root traits	↓ selected metals	Significant nutrient and plant responses
CCH6	↑ AP, AK, AH-N and SOM	↑ growth and root traits	↓ selected metals	Significant nutrient and plant responses
CCH8	↑ AP, AK, AH-N and SOM	↑ growth and root traits	↓ selected metals	Higher nutrient levels and plant performance than CK
CSH2	↑ AP, AK, AH-N and SOM	↑ growth and root traits	↓ selected metals	Significant nutrient and plant responses
CSH4	↑ AP, AK, AH-N and SOM	↑ growth and root traits	↓ selected metals	Significant nutrient and plant responses
CSH6	↑ AP, AK, AH-N and SOM	↑ growth and root traits	↓ selected metals	Significant nutrient and plant responses
CSH8	↑ AP, AK, AH-N and SOM	↑ growth and root traits	↓ selected metals	Higher nutrient levels and plant performance than CK
RHH2	↑ AP, AK and SOM; AH-N ≈ CK	↑ growth and root traits	↓ selected metals	AP, AK and SOM > CK; AH-N ≈ CK
RHH4	↑ AP, AK, AH-N and SOM	↑ growth and root traits	↓ selected metals	Significant nutrient and plant responses
RHH6	↑ AP, AK, AH-N and SOM	↑ growth and root traits	↓ selected metals	High plant performance across multiple traits
RHH8	Highest overall soil response within the tested range	Highest overall ryegrass performance	↓ selected metals	Highest means for all seven plant traits; Tukey group a for most or all traits

Overall statistical evidence for Panel A: Treatment significantly affected all seven ryegrass growth and root traits ($P < 0.001$). Significant treatment × time interactions were detected for pH and EC (both $P < 0.001$). Detailed nutrient, plant, and metal comparisons are reported in Tables S2–S7 and S21–S24 and S29–S32.

Panel B. Rhizobox experiment with alfalfa

**Treatment**	**Major soil responses**	**Soil structure and water properties**	**Alfalfa response**	**Metal response**	**Key statistical evidence**
CK	Reference	Reference	Reference	Reference	Reference
S	↑ nutrient status	↑ selected physical properties	↑ maximum root length	Reference for amendment comparisons	Maximum root length: Tukey d vs CK e
S-CSH	↑ AH-N, AP, AK and SOM	↑ dry-sieved aggregates and water properties	↑ maximum root length	↓ selected soil and plant metals vs S	Maximum root length: Tukey c
S-RHH	↑ AH-N, AP, AK and SOM	↑ dry-sieved aggregates and water	↑ maximum root length	↓ selected soil and plant metals	Maximum root length: Tukey b

		properties		vs S	
S-RHH-AMF	Highest AH-N, AP, AK and SOM[1]	Highest dry-sieved ≥0.5 mm fraction and all four water properties[1]	Greatest maximum root length	Further ↓ selected plant metals vs S-RHH	Nutrients: Tukey a[1]; water properties: Tukey a[1]; Maximum root length: Tukey a

[1] Values refer to the 0–10 cm soil layer at Month 4. S-RHH-AMF was significantly higher than the other treatments for AH-N and AK; it was also higher than S-RHH for AP and SOM ($P < 0.001$). For Sat.WC, FC, TP, and CP, the treatment ranking was S-RHH-AMF > S-RHH > S-CSH > S > CK, with distinct Tukey groups (a–e). The water-stable ≥0.5 mm aggregate fraction followed a different treatment ranking and was therefore not included in the "highest" designation.

Note: ↑ and ↓ indicate significant increases and decreases, respectively, relative to the corresponding reference treatment; ≈ indicates no significant difference ($P \geq 0.05$). CK was the reference for the pot experiment and S for amendment comparisons in the rhizobox experiment. Only major responses are summarized; complete statistical results are provided in Tables S2–S34. CCH, corncob hydrochar; CSH, corn straw hydrochar; RHH, rice husk hydrochar; AMF, arbuscular mycorrhizal fungi; AP, available phosphorus; AK, available potassium; AH-N, alkali-hydrolyzable nitrogen; SOM, soil organic matter; Sat.WC, saturated water content; FC, field capacity; TP, total porosity; CP, capillary porosity.
